# The efficacy of ginseng-containing traditional Chinese medicine in patients with acute decompensated heart failure: A systematic review and meta-analysis

**DOI:** 10.3389/fphar.2022.1083001

**Published:** 2023-01-10

**Authors:** Xiaozhe Chen, Yulong Ma, Jianhua Li, Lei Yao, Mingtai Gui, Bo Lu, Xunjie Zhou, Mingzhu Wang, Deyu Fu

**Affiliations:** Department of Cardiology, Yueyang Hospital of Integrated Traditional Chinese and Western Medicine, Shanghai University of Traditional Chinese Medicine, Shanghai, China

**Keywords:** acute decompensated heart failure, ginseng-containing traditional Chinese medicine, left ventricular ejection fraction, heart function, quality of life, adverse reactions and cases

## Abstract

**Objective:** To evaluate the efficacy of ginseng-containing traditional Chinese medicine (TCM) for acute decompensated heart failure (ADHF).

**Methods:** Seven databases were included from establishment until 10 July 2022. Pooled data were analyzed with random-effects model. The risk of bias was measured by the risk of bias tool for randomized trials (RoB 2). Modified Jadad scale score was used to assess the quality of including studies. The meta-analysis was performed with RevMan 5.3. Trial sequential analysis was assessed to avoid type I errors. We have registered our protocol in PROSPERO (CRD42021267742).

**Results:** Twenty-eight articles were included. The results demonstrated that compared with conventional western therapy (WT), ginseng-containing TCM combined with WT further improved clinical efficacy (RR: 1.25, 95% CI: 1.20–1.29, *p* < 0.00001, I^2^ = 8%), left ventricular ejection fraction (LVEF) (MD: 5.80, 95% CI: 4.86–6.74, *p* < 0.00001, I^2^ = 89%), stroke volume (MD: 13.80, 95% CI: 12.66–14.95, *p* < 0.00001, I^2^ = 93%), 6-min walk test (MD: 53.03, 95% CI: 20.76–85.29, *p* = 0.001, I^2^ = 97%), decreased 6-month rehospitalization (RR: 0.44, 95% CI: 0.18–1.11, *p* = 0.08, I^2^ = 0%), brain natriuretic peptide (MD: 188.12, 95% CI: 248.13 to -128.11, *p* < 0.00001, I^2^ = 94%), N-terminal pro-B-type natriuretic peptide (MD = -503.29; 95% CI: 753.18 to -253.40, *p* < 0.0001, I^2^ = 89%) and Minnesota living heart failure questionnaire scores (MD: 9.68, 95% CI: 13.67 to -5.70, *p* < 0.00001, I^2^ = 83%). The ROB2 assessment and modified Jaded scores showed most studies included were with some concerns.

**Conclusion:** Compared with WT alone, ginseng-containing TCM is a possible way to benefit ADHF patients. However, limited by the quality of including trials, more high-quality studies are needed to provide reliable evidence.

## 1 Introduction

Acute decompensated heart failure (ADHF), associated with poor prognosis and progressive multi-organ failure, is a fatal disease and a common cause of hospitalization worldwide (Desai and Stevenson, 2012; Verbrugge et al., 2020). One in six patients with heart failure dies within 30 days of hospitalization accounting for ADHF cases. Over one million patients with heart failure are hospitalized each year for ADHF in the United States and Europe (Benjamin et al., 2017). The pathophysiology of ADHF is multifactorial with potential precipitating factors (Njoroge and Teerlink, 2021). Patients with ADHF have a low quality of life and poor prognosis, accompanied by distressing symptoms of congestion and a risk of end-organ damage. Clinical trials have been conducted to treat the underlying cause and relieve symptoms; however, no treatment for ADHF has been shown to prolong survival or reduce morbidity (O’Connor et al., 2011; Chen et al., 2013; Ong et al., 2016).

Traditional Chinese medicine (TCM) has proven effective in oncology, respiratory, and neurology, with high patient compliance and few side effects reported (Chan et al., 2011; Jiao et al., 2017). Ginseng (*Panax ginseng C.A. Mey*) is a popular botanical drug that has been widely used to treat cardiovascular diseases. The use of ginseng in treating ADHF has a long history. Ginseng has been earlier reported to stop palpitations and calm the mind in the *Divine Husbandman’s Classic of the Materia Medica (Shennong Bencao Jing)*. In recent years, studies have shown that ginseng can maintain vascular tone, vasomotor function, balance blood pressure, and vascular endothelial functions (Irfan et al., 2020; Kim, 2018; Zhang, H., et al., 2020b), which benefit patients with ADHF. Two types of ginsengs are commonly used: white and red ginseng. Red ginseng is generally prepared by steaming it at 95°C–100°C for 2–3 h and then drying. In contrast, white ginseng is manufactured by sun-drying fresh ginseng, which causes a slight difference in their compositions and effects. Ginsenosides Rb1, Rb2, Rc, Rd, Rg1, and Re are the major constituents of ginseng, while Rg3, Rg5, Rg6, Rh1, Rh2, Rk1, Rs3, and F4 are unique constituents of red ginseng. Both types of ginsengs reinforce vital energy; however, the red ginseng is warm and known for homeostasis, while the white ginseng is mild and known for nourishing yin and tonifying the spleen and lungs. Both ginseng types are effective in treating cardiovascular diseases (Zhang et al., 2012).

Therefore, in China, ginseng-containing TCM is often used as adjunctive therapy for ADHF. However, the effects of ginseng on ADHF have not yet been systematically assessed. Therefore, this study aimed to review and compare the efficacy of combining ginseng-containing TCM *versus* Western therapy (WT) in treating ADHF.

## 2 Materials and methods

The Cochrane handbook for systematic reviews of interventions (Shamseer et al., 2015) and systematic review and meta-analysis protocol (Preferred Reporting Items for Systematic Reviews and Meta-Analyses; PRISMA) were used to prepare this review, as detailed in Supplementary Table S1. Additionally, we have registered our protocol in PROSPERO (CRD42021267742).

### 2.1 Search strategy

Search strategy was prepared according to reviews (Sun et al., 2016; Wang, 2016), expert consensus (Chen K. et al., 2016), and published meta-analyses (Wang, 2019; Lee et al., 2020), listed in Supplementary Table S2. Seven databases, including PubMed, EMBASE, Cochrane central register of controlled trials, China national knowledge infrastructure, Wanfang databases, Chongqing VIP, and Sinomed, were searched from their establishment until 10 July 2022. The following search terms were used (Acute Heart Failure [medical subject heading (MeSH)] OR Acute Decompensated Heart Failure OR cardiac failure OR myocardial failure OR heart decompensation OR CHF OR HF OR ventricular dysfunction) AND (Ginseng* OR Panax* OR jen shen* OR shen* jen). The detailed strategy was listed in the supplementary material. Ongoing trials are also listed in Supplementary Table S3.

### 2.2 Inclusion and exclusion criteria

Inclusion criteria were as follows:

1) Type of study: only randomized controlled trials (RCTs) were included. The RCTs should be written in English or Chinese.
2) Patients aged over 18 years with ADHF based on the guidelines for the diagnosis and treatment of heart failure (Zhang and Zhang, 2014; Heart Failure Group of Chinese Society of Cardiology of Chinese Medical Association et al., 2018; Wang and Liang, 2018).
3) The control group involved patients treated with only WT (including angiotensin-converting enzyme inhibitors, angiotensin receptor blockers, and diuretics) or with strong cardiac emergency drugs, such as dobutamine, if necessary.
4) The treatment group involved patients treated with all types of ginseng-containing TCM, including injection, tablets, granules, and decoction.
5) The outcomes included efficacy, left ventricular ejection fraction (LVEF), mortality, rehospitalization, left ventricular end-diastolic diameter (LVEDD), left ventricular end-diastolic volume (LVEDV), stroke volume (SV), brain natriuretic peptide (BNP), N-terminal pro-B-type natriuretic peptide (NT-proBNP), adverse reaction, 6-min walk test (6-MWT), and Minnesota living heart failure questionnaire (MLHFQ). At least one of these outcomes was included in this study.
6) When duplicate published studies were identified, we choose the most comprehensive report.

Exclusion criteria were as follows:

1) ADHF due to pulmonary heart disease, acute heart attack, or surgical causes.
2) Patients with severe hepatic or renal function damage.
3) Control group treated with Chinese herbal therapy.

### 2.3 Study selection and data extraction

Two reviewers selected the data sequentially in accordance with the inclusion and exclusion criteria. In cases of disagreements, a third reviewer was consulted. After selection, we listed the characteristics of the included RCTs, including the first author, country, age and sex of the participants, sample size, intervention, treatment duration, LVEF values, New York heart association grades, and outcomes. Finally, a third reviewer adjudicated any trial where there was disagreement regarding data extraction through discussion and consensus.

### 2.4 Risk of bias and quality assessment

Two reviewers individually evaluated the risk of bias for each study based on the description in the Cochrane handbook for systematic reviews of interventions (Cumpston et al., 2019). The Cochrane risk of bias tool for randomized trials (RoB 2) was used. The assess tool was ROB2_IRPG_beta_v7(Cochrane). Each included study was assessed in five domains including randomization process, deviations from intended interventions, missing outcome data, measurement of the outcome and selection of the reported result. We included the risk of bias in the characteristics table. In cases of disagreement in any study, a third reviewer adjudicated it.

What’s more modified Jadad scale was used to assess the study quality (Clark HD et al., 1999). Randomization, allocation hiding, blinding, withdrawais and dropouts were four domains in the assessment. The total score of the Jadad is 7. When score is 4–7, the study is a high-quality RCT, and when score is 1–3, the study was low quality.

### 2.5 Outcome definition

Outcomes were set according to the Expert Consensus on the Integrated Chinese and Western Medicine Treatment of chronic heart failure (Chen K. et al., 2016), the Chinese heart failure diagnosis and treatment guidelines 2018 (Wang and Liang, 2018), and the development of a core outcome set for the benefits and adverse events of acute heart failure in clinical trials of TCM and Western medicine: a study protocol (Qiu et al., 2021).

Efficacy and LVEF were the main outcomes. Efficacy was evaluated with heart function efficiency standard (HFES) or Chinese syndrome score (CSS) in reference to “the principle of clinical research on treating heart failure with new Chinese Medicine” (Zheng, 2002). Secondary outcomes included mortality, rehospitalization, heart structure, heart function, adverse events, and quality of life. Heart structure parameters included the LVEDD, LVEDV, and SV, while heart function parameters included BNP and NT-proBNP levels. Quality of life was assessed using the MLHFQ and 6-MWT. Data on readmissions, dyspnea scores, and edema scores were also collected, although they were rarely described in the literature.

### 2.6 Data analysis

The Mantel–Hanzal approach was applied to analyze binary variables using risk ratio (RR) with a 95% confidence interval (CI). The mean difference (MD) or standard mean difference with 95% CI was determined for continuous variables. All data were analyzed using a random-effects model. The I^2^ test and Chi-squared test were used to examine heterogeneity. The significance of the outcomes was determined using *p* values with statistical significance set at *p* < 0.05. The source of clinical heterogeneity, such as treatment duration, drug types, and LVEF values, were calculated using subgroup analysis. Sensitivity analysis was performed by deleting each trial to ensure the results were stable. Egger’s tests were used to examine potential publication bias when studies were >10.

We used trial sequential analysis (TSA) to prevent type I errors and repeated significance tests due to small data sizes and meta-analysis (Brok et al., 2008). The required information size (RIS) was estimated and adjusted using TSA-evaluated meta-analysis diversity. The TSA required type I and II errors for completion. The RIS was calculated in this study using *α* = 0.05 (two-sided), with an 80% power level.

## 3 Results

### 3.1 Search results

**FIGURE 1:**
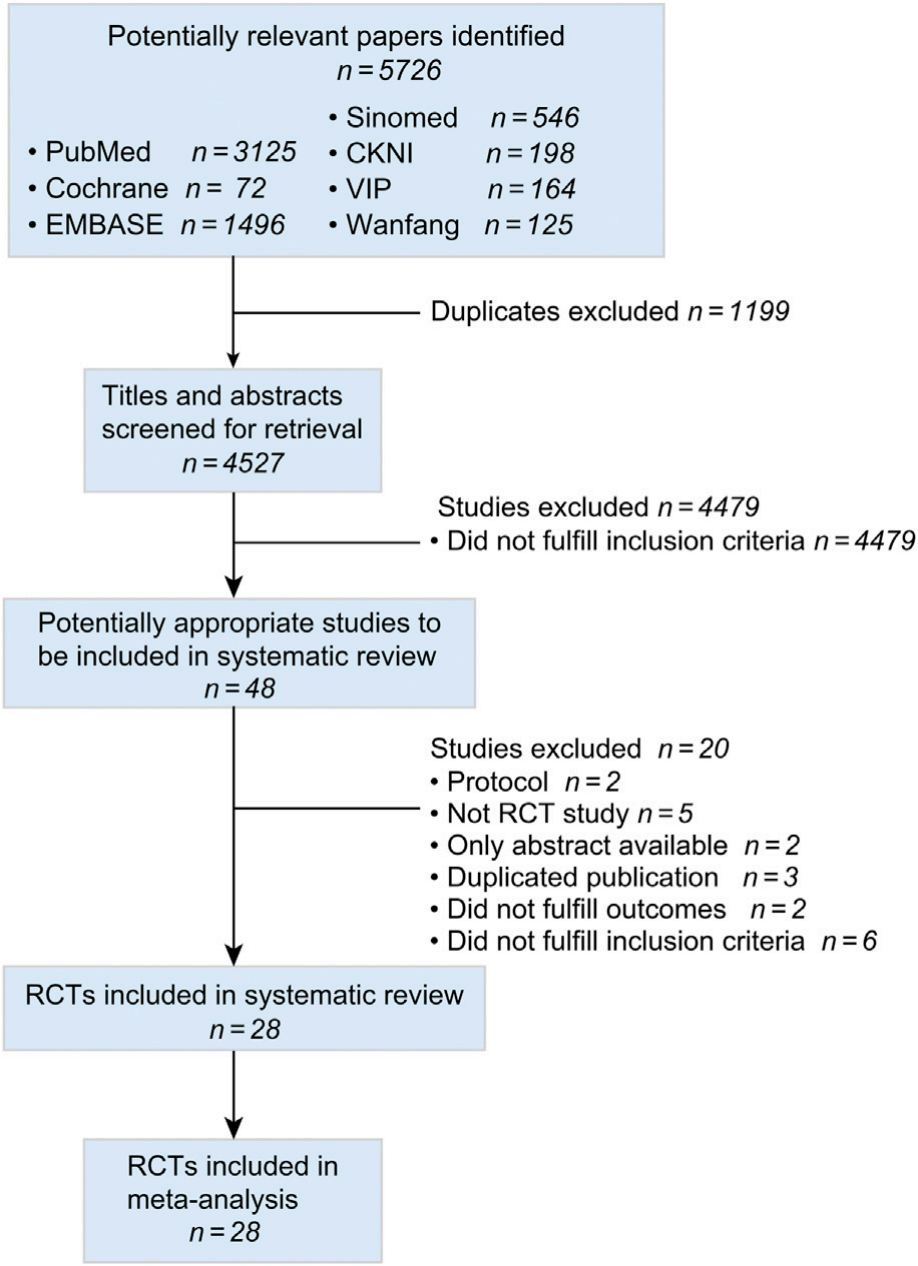
The PRISMA study flowchart of study search.

Figure 1 was a flowchart of the search results according to the PRISMA guideline. We included 5,726 records from seven databases. After removing duplicate studies, we screened 4,527 titles and abstracts, obtaining 48 full-text articles. Twenty studies were excluded including two protocols (ClinicalTrials.gov, 2014; Wang et al., 2018), five non-RCT studies (Chen, 2015; J. X. and Ma, 2017; Zhen et al., 2020; Ye et al., 2021; Zhang, 2020), two studies with only abstracts (Zhang, 2017; Yang et al., 2020), three duplicated publicized studies (Zhang, 2007; Hao, 2009; Yuan et al., 2019), two studies that did not fulfil outcomes (Ruan et al., 2014; Lei and Zhang, 2016), and six studies that did not fulfil inclusion criteria (Xu, 1998; Yuan, 1998; Chen and Li, 2009; Luo et al., 2015a; An et al., 2017; Wang, 2017). Finally, 28 articles were included (Ai and Xu, 2019; Chang and Yang, 2015; Deng, 2016; Feng, 2019; Fu et al., 2021; Huang et al., 2019; Huang, 2006; Li et al., 2015; Li et al., 2017; Luo et al., 2015b; Mao, 2018; Pan, 2014; Ran, 2017; Sun et al., 2019; Sun, 2016; Wang et al., 2019; Xiong and Zhou, 2009; Ye et al., 2007; Zhang et al., 2006; Zhang, 2018; Zhan and Hu, 2013; Zhang, Y., et al., 2020a; Zhao et al., 2014; Zhao, 2014; Zhen et al., 2014; Qin et al., 2019; Jia, 2021; P. X., 2019).

### 3.2 Study characteristics

Basic information (study design, LVEF value, patients’ age) of the 28 papers were included (Table 1). All studies were RCTs, one was designed with three arms (Zhao, 2014) and one with four arms (Sun et al., 2019). All studies were conducted in China. One study was written in English (Wang et al., 2019) and the others were written in Chinese. The sample size ranged from 10 participants per arm to 150 participants per arm. Ages ranged from 43.68 ± 16.98 to 70.21 ± 2.34 years and 41.68 ± 17.14 to 70.30 ± 15.00 years in the intervention and control groups, respectively. Five ginseng-containing TCMs were included. Twelve studies were interacted with Shenfu injection (SFI) (Zhao, 2014; Zhao et al., 2014; Luo et al., 2015b; Deng, 2016; Sun, 2016; Li et al., 2017; Ran, 2017; Mao, 2018; Zhang, 2018; Huang et al., 2019; Qin et al., 2019; Wang et al., 2019). Nine studies were interacted with Shenmai injection (SMI) (Feng, 2019; Fu et al., 2021; Huang, 2006; Li et al., 2015; Pan, 2014; Ye et al., 2007; Zhang and Hu, 2013; Zhang, Y., et al., 2020a; Zhen et al., 2014). Four studies were interacted with Shengmai injection (SGMI) (Chang and Yang, 2015; Xiong and Zhou, 2009; Zhang et al., 2006; P. X., 2019). One study was interacted with Yangxinshi tablets (YXST) (Sun et al., 2019), and two studies used Qili Qiangxin (QLQX) tablets (Ai and Xu, 2019; Jia, 2021). The detailed compositions of the ginseng-containing TCMs are listed in Table 2. The names of the botanical drugs were standardized by the Kew Medicinal Plant Names Services (MPNS). However, most botanical drugs are protected by patents, so we do not have access to information on ingredient dosages and extraction processes. What’s more we also listed the source, quality control and chemical analysis of the including botanical drug in Table 3 according to the guidelines on medicinal plants and extracts (Heinrich M et al., 2022). We found that all included studies did not mention the quality control and chemical analysis in the trials.

**TABLE 1:** Details of 28 papers included in the meta-analysis.

Study	LVEF	Interaction group						Control group				Outcomes
		Sample size	Age	Female	Intervention	Drug delivery	Frequency/day/interval	Sample size	Age (year)	Female	Control	
Zhang, D	not limited	24	68.5 ± 11.8	45.80%	SGMI + WT	injection	Qd/7/60 mL	22	70.30 ± 15.00	36.40%	WT + Dobutamine	1,4,13
Huang, Z	not limited	25	61.25 ± 5.65	56.00%	SMI + DZHS + WT	injection	Qd/10/30 mL	25	57.45 ± 6.35	52.00%	WT	1,4,5,7,13
Ye, C	not limited	50	64.35 ± 5.62	46.00%	Dobutamine + SMI + WT	injection	Qd/10/30 mL	50	63.73 ± 3.85	52.00%	WT	1,4,7,13
Xiong, M	not limited	64	unclear	46.90%	SGMI + Xiangdan injection + WT	injection	Qd/7/40 mL	60	unclear	46.67%	WT	1,4,13
Zhang, M	not limited	42	66.2 ± 11.5	52%	SMI + WT	injection	Qd/7d/20–30 mL	42	65.6 ± 11.2	57%	WT	1,5,13,14
*Pan*, X	<40	20	65.8 ± 2.4	unclear	Zuosimendan + SMI + WT	injection	Qd/7/40 mL	20	66.4 ± 7.9	unclear	WT + Zuosimendan	3,4,5,7,10,13
Zhao, H	not limited	21	66.6 ± 7.1	unclear	SFI + WT	injection	Qd/14/80 mL	21	65.8 ± 6.9	unclear	WT	1,4,6,9,13
Zhao, W	not limited	21	67.57 ± 11.48	52.38%	SFI + WT	injection	Qd/5–10/50 mL	21	61.1 ± 7.5	75.80%	WT	1,5,9,12,13
	not limited	21	64.14 ± 8.86	47.62%	SFI + WT	injection	Qd/5–10/100 mL					
Zhen, Y	<45	36	64.75 ± 8.33	52.78%	SMI + Honghuahuang injection + WT	injection	Qd/10/100 mL	36	65.20 ± 9.15	50	WT	1,2,4,5,6,10,13
Li, L	<45	30	58.25 ± 7.84	43.30%	SMI + WT	injection	Qd/14/100 mL	30	57.82 ± 7.26	53.33	WT	1,2,11,12,13
Luo, B	<50	24	53.40 ± 11.70	41.67	SFI + WT	injection	Qd/7/50 mL	24	50.9 ± 12.5	37.50%	WT	2,4,5,10,13
Chang, R	not limited	60	70.21 ± 2.34	45.00%	Dobutamine + SGMI + WT	injection	Qd/14/60 mL	60	69.97 ± 3.47	46.67%	WT + Dobutamine	1,4,7,13
Deng, J	≤40	30	68.67 ± 6.90	50.00%	SFI + WT	injection	Qd/7/80 mL	30	68.63 ± 6.65	46.67%	WT	4,5,9,13
Sun, Y	not limited	120	62 ± 2.16	40.00%	SFI + WT	injection	10 times a month/180/60 mL	120	61.0 ± 2.43	36.67%	WT	1,11,13
Li, Z	<35	50	60.20 ± 13.91	32.00%	SFI + WT	injection	Qd/10–14/80 mL	50	59.73 ± 14.82	38.00%	WT	1,4,5,8,9,11,13
Ran, Y	not limited	20	69.70 ± 9.10	45.00%	SFI + WT	injection	Qd/14/50 mL	20	69.9 ± 9.0	40.00%	WT	1,2,4,5,6,10,12,13
Mao, X	not limited	48	68.43 ± 9.22	unclear	SFI + WT	injection	Qd/14/100 mL	48	69.20 ± 9.17	45.65%	WT	1,4,8,13
Zhang, J	<40	59	57.85 ± 14.38	33.59%	SFI + RHBNP + WT	injection	Qd/3/50–100 mL	58	58.43 ± 13.26	37.90%	WT + RHBNP	3,4,5,6,9,13
Wang, X	<50	80	68.58 ± 8.42	43.24%	SFI + WT	injection	Qd/7/50 mL	80	68.14 ± 8.73	31.43%	WT + GS150	1,2,3,4,5,11,13
Feng, Z	not limited	56	58.80 ± 6.50	48.21	SMI + WT	injection	Qd/14/100 mL	56	59.50 ± 6.80	46.43%	WT	1,6,11,12,13
Sun, F	not limited	30	62.23 ± 10.83	23.33%	YXST + WT	orally	Tid/180/3#	30	62 ± 10.7	30.00%	WT	4,5,6,7,8,11,12,13,14
	not limited	10	63.30 ± 8.74	40.00%	YXST + WT	orally	Tid/180/3#	10	61.8 ± 8.6	66,66%	WT + Trimetazidine	
AI, K	not limited	150	67.69 ± 10.54	47.3	QLQX + WT	orally	Tid/12/4#	150	68.37 ± 12.63	46.00%	WT	1,4,6,7,9,13
Huang, X	<40	40	65.5 ± 3.7	44%	Nitroglycerin + SFI + WT	injection	Qd/7/50 mL	40	68.2 ± 9.1	50.00%	WT + Milrinone	1,4,5,6,8,11,10,13
Peng. (2019)	not limited	62	43.68 ± 16.98	61.29%	SGMI + RHBNP + WT	injection	Qd/7/25–50 mL	62	41.68 ± 17.14	66.13%	WT + RHBNP	3,4,5,10,13
Qin,G	not limited	75	65.3 ± 9.8	40.00%	SFI + Zuosimenda + WT	injection	Qd/14/100 mL	75	66.0 ± 10.1	40.67%	WT + Zuosimenda	4,6,9,13
Zhang, Y	<45	30	64.12 ± 6.13	30.00%	SMI + ENN + WT	injection	QD/14/50 mL	30	62.89 ± 5.74	40.00%	ENN	1,4,5,6, 10,11,12,13
Fu, X	not limited	55	59.33 ± 10.09	38.18%	SMI + RHBNP + WT	injection	Qd/3/50 mL	55	60.21 ± 8.12	36.40%	WT + RHBNP	1,2,4,5,6,10,13
Jia, Y	≤40	60	64.89 ± 6.54	43.33%	QLQX + rhBNP + WT	orally	Tid/28/4#	60	65.22 ± 6.79	41.66%	WT + rhBNP	1,4,13

Outcomes: 1. HFES, 2; CSS, 3. Other Effective Measurement, 4. LVEF, 5. Adverse Reaction and Adverse Cases, 6. LVEDD, 7; SV, 8; LVEDV, 9. NT-proBNP, 10; BNP, 11.6MWT, 12; MLFHQ, 13. Mortality, 14. Rehospitalization.

**TABLE 2:** Five ginseng-containing TCM included in the meta-analysis.

Formulation	Combination
YXST	*1*) *Astragalus mongholicus Bunge* [Fabaceae; Astragali mongholici radix]
	*2*) *Codonopsis pilosula (Franch.) Nannf.* [Campanulaceae; codonopsis radix]
	*3*) *Salvia miltiorrhiza Bunge* [Lamiaceae; Salviae miltiorrhizae radix et rhizoma]
	*4*) *Pueraria montana* var. *Lobata (Willd.) Maesen and S.M.Almeida ex Sanjappa and Predeep* [Fabaceae; puerariae lobatae radix]
	*5*) *Epimedium sagittatum (Siebold and Zucc.) Maxim.* [Berberidaceae; epimedii folium]
	*6*) *Crataegus pinnatifida Bunge* [Rosaceae; crataegi folium]
	*7*) *Rehmannia glutinosa (Gaertn.) DC.* [Orobanchaceae; radix rehmanniae preparata]
	*8*) *Angelica sinensis (Oliv.) Diels* [Apiaceae; angelicae sinensis radix]
	*9*) *Coptis chinensis Franch.* [Ranunculaceae; coptidis rhizoma]
	*10*) *Corydalis yanhusuo (Y.H.Chou and Chun C.Hsu) W.T.Wang ex Z.Y.Su and C.Y.Wu* [Papaveraceae; corydalis rhizoma]
	*11*) *Sesamum indicum L.* [Pedaliaceae; schizonepetae spica]
	*12*) *Panax ginseng C.A.Mey.* [Araliaceae; folium ginseng]
	*13*) *Glycyrrhiza uralensis Fisch. ex DC.* [Fabaceae; extractum glycyrrhizae]
QLQX	*1*) *Astragalus mongholicus Bunge* [Fabaceae; Astragali mongholici radix]
	*2*) *Panax ginseng C.A.Mey.* [Araliaceae; folium ginseng]
	*3*) *Aconitum carmichaeli Debeaux* [Ranunculaceae; aconiti lateralis radix praeparata]
	*4*) *Salvia miltiorrhiza Bunge* [Lamiaceae; Salviae miltiorrhizae radix et rhizoma]
	*5*) *Descurainia sophia L.) Webb ex Prantl* [Brassicaceae; descurainiae semen, lepidii semen]
	*6*) *Alisma plantago-aquatica subsp. Orientale (Sam.) Sam.* [Alismataceae; alismatis rhizoma]
	*7*) *Polygonatum odoratum (Mill.) Druce* [Asparagaceae; polygonati odorati rhizoma]
	*8*) *Neolitsea cassia L.) Kosterm.* [Lauraceae; chinese cinnamon]
	*9*) *Carthamus tinctorius L.* [Asteraceae; carthami flos]
	*10*) *Periploca sepium Bunge* [Apocynaceae; acanthopanacis cortex (periploca sepium)]
	*11*) *Citrus × aurantium L.* [Rutaceae; aurantii amari epicarpium et mesocarpium]
SFI	*1*) *Panax ginseng C.A.Mey* [Araliaceae; folium ginseng]
	*2*) *Aconitum carmichaeli Debeaux* [Ranunculaceae; aconiti lateralis radix praeparata]
SMI	*1*) *Panax ginseng C.A.Mey.* [Araliaceae; folium ginseng]
	*2*) *Ophiopogon japonicus (Thunb.) Ker Gawl.* [Asparagaceae; liriopis tuber]
SGMI	*1*) *Panax ginseng C.A.Mey.* [Araliaceae; folium ginseng]
	*2*) *Schisandra chinensis (Turcz.) Baill.* [Schisandraceae; fructus schisandrae]
	*3*) *Ophiopogon japonicus (Thunb.) Ker Gawl.* [Asparagaceae; liriopis tuber]

**TABLE 3:** The source, quality control and chemical analysis of five ginseng-containing TCM.

Formulation	Study	Source	Quality control reported	Chemical analysis
YXST	Sun et al. (2019)	SPH Qingdao Growful Pharmaceutical Co., Ltd.	No	No
QLQX	Ai and Xu (2019); Jia, (2021)	SHIJIAZHUANG YILING PHARMACEUTICAL CO., LTD.	No	No
SFI	Deng, 2016; Huang et al., 2019; Luo et al., 2015a; Mao, 2018; Ran, 2017; Zhao et al., 2014; Qin et al., 2019	Ya’an Sanjiu Chinese Medicinal Materials Technology Industrialization Co., Ltd.	No	No
	Zhao H, (2014)	Shenzhen China Resources Sanjiu Medicine Trading CO., LTD.	No	No
	Sun, 2016; Li et al., 2017; Wang et al., 2018; Zhang, 2018	not mention	No	No
SMI	Pan, (2014)	Ya’an Sanjiu Chinese Medicinal Materials Technology Industrialization Co., Ltd.	No	No
	Zhang and Hu, 2013; Zhang, Y., et al., 2020a	CHINA (HANGZHOU) QING CHUN BAO GROUP CO., LTD.	No	No
	Zhen et al. (2014)	not mention	No	No
	Feng, 2019; Huang, 2006; Li et al., 2015; Ye et al., 2007; Zhen et al., 2014	Shineway Pharmaceutical Group (Shandong) Co., Ltd	No	No
	Fu et al. (2021)	DALI Pharmaceutical Group Co., Ltd	No	No
SGMI	Chang and Yang, (2015)	Shijiazhuang Taihang Pharmaceutical Co., Ltd	No	No
	Xiong and Zhou. 2009	not mention	No	No
	Zhang et al. (2006)	Ya’an Sanjiu Chinese Medicinal Materials Technology Industrialization Co., Ltd.	No	No
	Pan. (2014)	Shandong Jiankang Yuan Co., Ltd.	No	No

Eleven studies (Pan, 2014; Zhen et al., 2014; Li et al., 2015; Luo et al., 2015b Deng, 2016; Li et al., 2017; Zhang, 2018; Huang et al., 2019; Wang et al., 2019; Fu et al., 2021) with limited EF values differed by 35%–50% from the other 17 studies without limitations. The duration of treatment differed by 3 days–180 days.

### 3.3 Risk of bias and quality assessment

**FIGURE 2:**
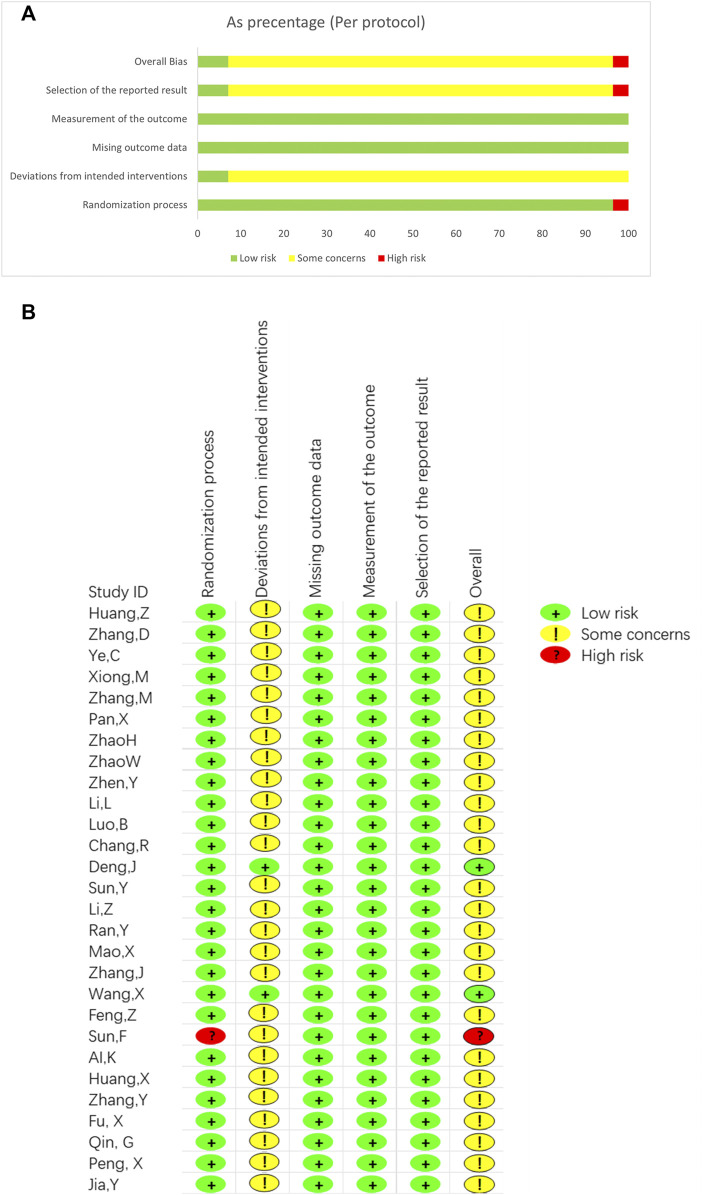
Risk of bias summary and diagram. **(A)** Risk of bias summary. **(B)** Risk of bias diagram.

Figure 2 depicts the risk of bias for all included studies, ranging from low to high. We also assess the quality of the studies with modified Jadad scores (Supplementary Table S5). The score ranged from one to 8. All 28 studies referred to randomization, in which 13 used a randomized number table (Ai and Xu, 2019; Feng, 2019; Fu et al., 2021; Li et al., 2015; Li et al., 2017; Zhang et al., 2006; Zhang, 2018; Zhang, 2020; Zhang, Y., et al., 2020a; Zhao, 2014; Zhen et al., 2014; Jia, 2021; P. X., 2019). One used a randomized program (Wang et al., 2019), 13 did not mention a randomized method, and one used admission time to randomize the number of participants with high risk (Sun et al., 2019). Two studies (Deng, 2016; Wang et al., 2019) mentioned blinding of placebo-treated patients, and one study mentioned blinding of the statisticians (Wang et al., 2019). The other 26 studies did not mention blinding methods and allocation hiding. Overall, two studies were low risk, and one study was of a high risk, the other 25 was of some concerns (Supplementary Table S4). According to the modified Jadad scores (Supplementary Table S5), 15 studies are low quality, and the lowest score is 1 (Sun et al., 2019). Allocation hiding is common in the low-quality study. Though the other 13 studies are with a high quality, most studies scored 4. One study scored 5 (Deng, 2016) and one study scored 7 (Wang et al., 2019). Overall, the quality of included studies was low. Randomised methods and blinding were the most significant factors limiting the quality of included studies.

### 3.4 Main outcomes

#### 3.4.1 Efficacy

**FIGURE 3:**
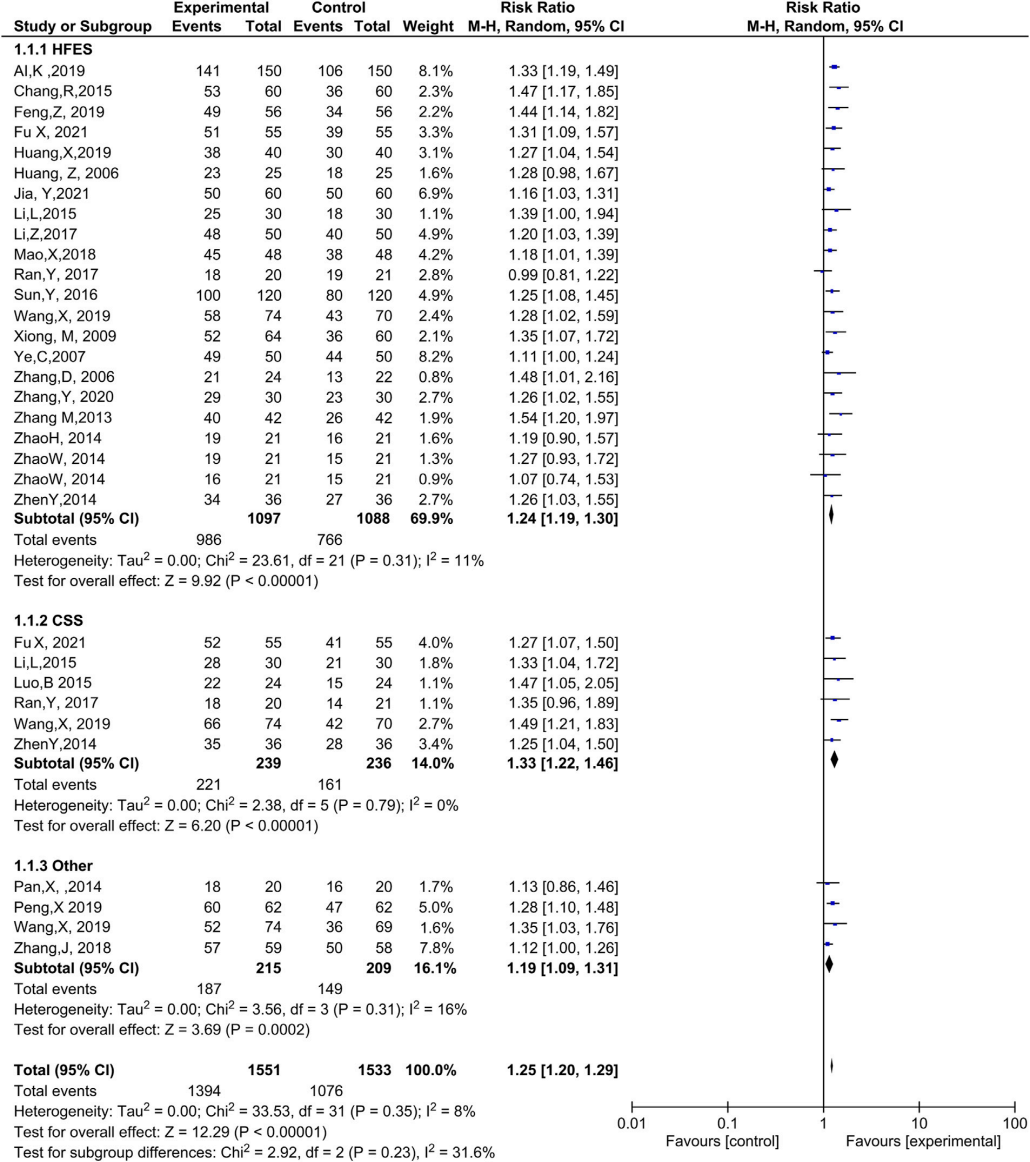
Meta-analysis results of efficacy between the experimental and control groups.

Twenty-one trials (Ai and Xu, 2019; Chang and Yang, 2015; Feng, 2019; Fu et al., 2021; Huang et al., 2019; Huang, 2006; Li et al., 2015; Li et al., 2017; Mao, 2018; Ran, 2017; Sun, 2016; Wang et al., 2019; Xiong and Zhou, 2009; Ye et al., 2007; Zhang et al., 2006; Zhang, 2020; Zhang, Y., et al., 2020a; Zhao et al., 2014; Zhao, 2014; Zhen et al., 2014; Jia, 2021) with 2,185 patients reported efficacy based on heart function. The meta-analysis discovered that ginseng-containing TCM improved the HFES with compelling homogeneity (RR: 1.24, 95% CI: 1.19–1.30, *p* < 0.00001, I^2^ = 11%, Figure 3).

Six trials (Zhen et al., 2014; Luo et al., 2015b; Li et al., 2015; Ran, 2017; Wang et al., 2019; Fu et al., 2021) with 475 cases reported CSS as an efficacy measurement. The results indicated that ginseng-containing treatment led to an increase in CSS (RR: 1.33, 95% CI: 1.22–1.46, *p* < 0.00001, I^2^ = 0%, Figure 3).

However, two articles (Pan, 2014; Zhang, 2018) did not mention an efficacy standard. One article measured efficacy using Lee’s heart failure standard (Wang et al., 2019), while another (P. X., 2019) measured efficacy using the European collaboration on ADHF. Compared with WT, ginseng-containing TCM improved clinical efficacy (RR: 1.19, 95% CI: 1.09–1.31, *p* = 0.0002, I^2^ = 16%, Figure 3). Therefore, ginseng-containing TCM could improve clinical efficacy in the HFES, CSS, and other measurements.

#### 3.4.2 LVEF

**FIGURE 4:**
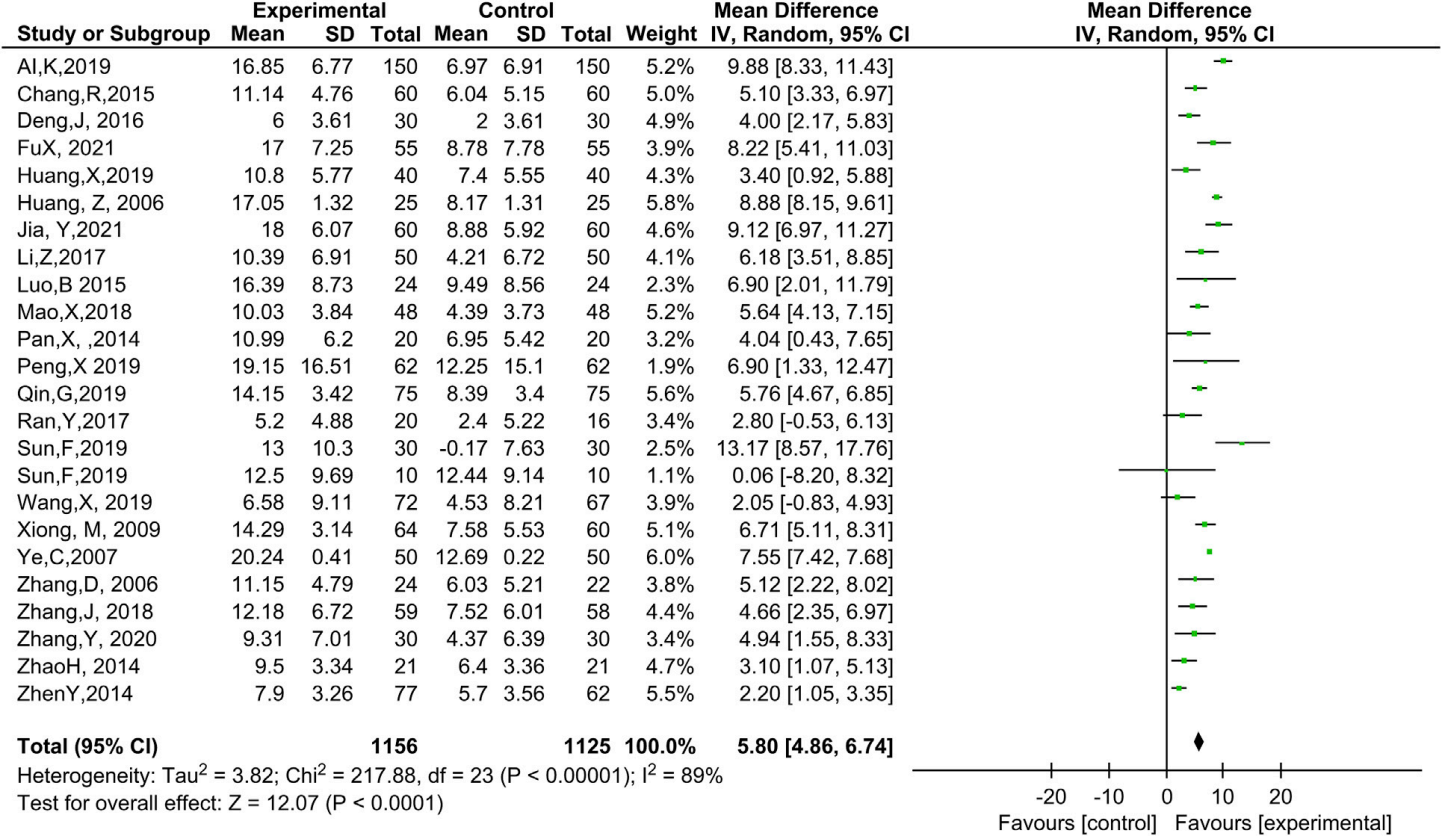
Meta-analysis results of LVEF between the experimental and control groupsL VEF, left ventricular ejection fraction.

Twenty-three studies (Ai and Xu, 2019; Chang and Yang, 2015; Deng, 2016; Fu et al., 2021; Huang et al., 2019; Huang, 2006; Li et al., 2017; Luo et al., 2015b; Mao, 2018; Pan, 2014; Ran, 2017; Sun et al., 2019; Wang et al., 2019; Xiong and Zhou, 2009; Ye et al., 2007; Zhang et al., 2006; Zhang, 2018; Zhang, Y., et al., 2020a; Zhao et al., 2014; Zhen et al., 2014; Qin et al., 2019; Jia, 2021; P. X., 2019) involving 2,281 patients included LVEF as an outcome (Figure 4). Compared with WT, the combination with ginseng-containing TCM led to an increase in LVEF values (MD: 5.80, 95% CI: 4.86–6.74, *p* < 0.00001, I^2^ = 89%).

### 3.5 Secondary outcomes

#### 3.5.1 Mortality and rehospitalization

All studies recorded mortality, with the control group recording only one death. Two studies recorded 6-month readmission rate (Sun et al., 2019; Zhang, 2020), indicating that ginseng-containing TCM could reduce 6-month rehospitalization (RR: 0.44, 95% CI: 0.18–1.11, *p* = 0.08, I^2^ = 0%, Supplementary Figure S1).

#### 3.5.2 Heart structure and function

Heart structure was measured using LVEDD, SV, and LVEDV. Heart function was measured based on BNP or NT-proBNP levels. Ten studies (Ai and Xu, 2019; Feng, 2019; Fu et al., 2021; Huang et al., 2019; Ran, 2017; Sun et al., 2019; Zhang, 2018; Zhang, Y., et al., 2020a; Zhao et al., 2014; Zhen et al., 2014) reported that ginseng-containing TCM reduced LVEDD (MD: 2.64, 95% CI: 4.39 to -0.89, *p* = 0.003, I^2^ = 87%) (Supplementary Figure S2). SV improved in six trials (Huang, 2006; Ye et al., 2007; Pan, 2014; Chang and Yang, 2015; Ai and Xu, 2019; Sun et al., 2019) with the use of ginseng-containing TCMs (MD: 13.80, 95% CI: 12.66–14.95, *p* < 0.00001, I^2^ = 93%) (Supplementary Figure S3). LVEDV improvement (Li et al., 2017; Mao, 2018; Huang et al., 2019; Sun et al., 2019) was reported in four studies (MD: 16.76, 95% CI: 25.71 to -7.80, *p* = 0.0002, I^2^ = 0%) (Supplementary Figure S4), indicating that ginseng-containing TCM substantially improved heart structure.

For heart function, eight trials (Fu et al., 2021; Huang et al., 2019; Luo et al., 2015b; Pan, 2014; Ran, 2017; Zhang, Y., et al., 2020a; Zhen et al., 2014; P. X., 2019) reported a decrease in BNP levels after treatment with ginseng-containing TCM (MD: 188.12, 95% CI: 248.13 to -128.11, *p* < 0.00001, I^2^ = 94%) (Supplementary Figure S5). Seven studies (Zhao, 2014; Zhao et al., 2014; Deng, 2016; Li et al., 2017; Zhang, 2018; Ai and Xu, 2019; Qin et al., 2019) reported NT-proBNP levels in 853 patients. Compared with pure WT, ginseng-containing TCM treatment reduced NT-proBNP levels in these patients (MD = -503.29; 95% CI: 753.18 to -253.40, *p* < 0.0001, I^2^ = 89%) (Supplementary Figure S6).

#### 3.5.3 Quality of life

Quality of life was measured using the 6-MWT and MLHFQ. According to the results, compared with WT, statistics showed an increase in 6-MWT (MD: 53.03, 95% CI: 20.76–85.29, *p* = 0.001, I^2^ = 97%) in eight included trials (Feng, 2019; Huang et al., 2019; Huang, 2006; Li et al., 2015; Sun et al., 2019; Sun, 2016; Wang et al., 2019; Zhang, Y., et al., 2020a) (Supplementary Figure S7). Six studies (Feng, 2019; Li et al., 2015; Ran, 2017; Sun et al., 2019; Zhang, Y., et al., 2020a; Zhao, 2014) including 436 patients reported MLHFQ results in their trial. The results demonstrated that ginseng-containing TCM treatment decreased MLHFQ scores (MD: 9.68, 95% CI: 13.67 to -5.70, *p* < 0.00001, I^2^ = 83%) (Supplementary Figure S8).

#### 3.5.4 Adverse reactions and cases

Sixteen studies documented adverse cases: six trials (Deng, 2016; Huang et al., 2019; Huang, 2006; Luo et al., 2015b; Sun et al., 2019; Zhang, 2020) reported no adverse events, and 10 documented adverse events (Fu et al., 2021; Li et al., 2017; Pan, 2014; Ran, 2017; Zhang, 2018; Zhang, 2020; Zhang, Y., et al., 2020a; Zhen et al., 2014; P. X., 2019). Thirteen adverse reactions and cases (erythema, headache, arrhythmia, dizziness, vomiting, nausea, red face, chest tightness, chills, liver dysfunction, renal dysfunction, hypokalemia, and low blood pressure) were recorded. Compared with pure WT, ginseng-containing TCM reduced the risk of dizziness (RR = 0.24; 95% CI: 0.08–0.67, *p* = 0.007, I^2^ = 0%) and did not increase the risk of other adverse reactions and cases, suggesting that ginseng-containing TCM is safe to use (Table 4).

**TABLE 4:** Adverse reactions and cases between the two groups.

Outcomes	Trials	Experimental group (events/total	Control group (events/total	Statistical method	RR, 95%Cl	I^2^	P
Erythra	3	2/167	4/166	REM	0.55 [0.11–2.60]	0	0.45
Headache	3	2/145	2/144	REM	0.99 [0.20–5.00]	0	0.99
Arrhythmia	3	0/92	3/92	REM	0.32 [0.05–2.09]	0	0.24
Dizziness	3	4/102	17/102	REM	0.24 [0.08–0.67]	0	0.007
Vomiting	2	5/92	10/92	REM	0.47 [0.15–1.44]	0	0.19
Nauseating	2	5/104	10/104	REM	0.47 [0.16–1.44]	0	0.19
Red face	1	1/20	0/20	-	-	-	-
Chest tightness	1	0/55	1/55	-	-	-	-
Chill	1	1/78	0/79	-	-	-	-
Liver dysfunction	3	2/167	4/166	REM	0.55 [0.11–2.60]	0	0.45
Renal dysfunction	3	8/170	8/170	REM	1 [0.37–2.73]	0	1
Hypokalaemia	2	2/56	0/56	REM	3.12 [0.31–30.98]	0	0.33
Low blood pressure	3	1/109	4/108	REM	0.39 [0.07–2.03]	0	0.26

### 3.6 Other outcomes

One study recorded the length of hospitalization (Zhao, 2014), one recorded the integration of dyspnea, edema, and pulmonary rales (Zhang and Zhang, 2014), one recorded the efficacy of dyspnea, edema, and pulmonary rales (Ai and Xu, 2019), and one recorded the physical fitness of patients (Zhang, Y., et al., 2020a).

### 3.7 Subgroup analysis

Since different interactions, treatment duration was performed in the test groups and control groups, a subgroup analysis was performed to eliminate the effects of confounding factors on the outcomes. Subgroup studies were conducted according to drug type, treatment duration, and LVEF values. As shown in Table 5 and Supplementary Table S5, the results of the subgroup analysis were consistent with the overall study results. After subgroup analysis, heterogeneity was significantly reduced for LVEF, suggesting that treatment duration, drug type, and LVEF may be the source of heterogeneity.

**TABLE 5:** Subgroup analysis of main outcomes.

Outcome or subgroup	Studies	Participants		MD/RR (95% CI)	Z	P	Heterogeneity	
							I^2^	P_h_
1. HFES	21	2,185		1.24 [1.19–1.30]	9.85	<0.00001	11	0.31
SFI	8	827		1.19 [1.12–1.28]	5.29	<0.00001	0	0.76
SGMI	3	290		1.43 [1.23–1.66]	4.62	<0.00001	0	0.86
QLQX	2	300		1.25 [1.08–1.43]	5.05	<0.00001	65	0.09
SMI	8	648		1.28 [1.17–1.41]	5.3	<0.00001	37	0.13
Treatment duration								
≤14 days	18	1,525		1.24 [1.18–1.31]	8.13	<0.00001	15	0.27
>14 days	3	660		1.25 [1.15–1.36]	5.09	<0.00001	28	0.25
LVEF								
LVEF ≤50%	6	556		1.22 [1.13–1.31]	5.27	<0.00001	0	0.87
LVEF not limited	15	1,629		1.26 [1.19–1.34]	7.55	<0.00001	30	0.13
2. LVEF	23	2,281		5.80 [4.86–6.74]	10.45	0.002	89	<0.00001
Different drugs								
SFI	10	868		4.27 [3.65–5.44]	8.93	<0.00001	42	0.08
SGMI	4	414		5.90 [4.83–6.98]	10.31	<0.00001	0	0.54
SMI	6	499		6.12 [4.22–8.02]	6.31	<0.00001	95	<0.00001
QLQX	2	420		9.62 [8.36–10.88]	15.02	<0.00001	0	0.57
YXST	1	80		7.08 [-5.73–19.90]	1.08	0.28	86	0.007
Treatment duration								
≤14 days	20	1781		5.24 [4.23–6.25]	10.13	<0.00001	90	<0.00001
>14 days	3	500		9.47 [7.02–11.92]	7.57	<0.00001	61	0.05
LVEF								
LVEF ≤50%	10	903		4.63 [3.08–6.19]	5.83	<0.00001	76	<0.0001
LVEF not limited	13	1,378		6.67 [5.72–7.62]	13.74	<0.00001	85	<0.00001

### 3.8 Sensitivity analysis

The main outcomes, encompassing the HFES and LVEF, were tested using a sensitivity analysis, which involved removing each trial to assess the robustness of the main outcome. The pooled RR values of the HFES and the MD values of the LVEF were stable, as shown in Supplementary Figures 9 and 10.

### 3.9 TSA for HFES and LVEF

**FIGURE 5:**
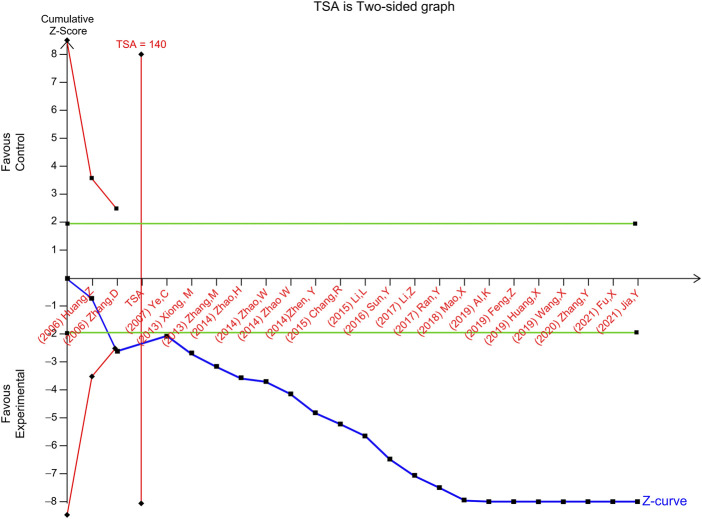
TSA of heart function efficacy standard TSA, trial sequential analysis.

TSA of the effect of ginseng-containing TCM *versus* WT on HFES was performed. In a random-effects model, the TSA-adjusted CI for the meta-analysis of HFES had an RR of 0.95 (95% CI, I^2^ statistic = 0%, diversity D^2^ = 0%). In the meta-analysis of ginseng-containing TCM efficacy, the point estimate of the possible intervention effect proposed by a trial with a low risk of bias (Wang et al., 2019) was a relative risk reduction of -27.59 percent, and the estimated RIS was 140 participants (Figure 5). The cumulative Z-curve (blue line) breaks through the conventional statistical boundary (green line), trial sequential monitoring boundary for benefit (red inward sloping line), and RIS boundary (red line) with an estimated value of 2,185 participants, indicating that sufficient information was applied to prove the reliability of HFES.

**FIGURE 6:**
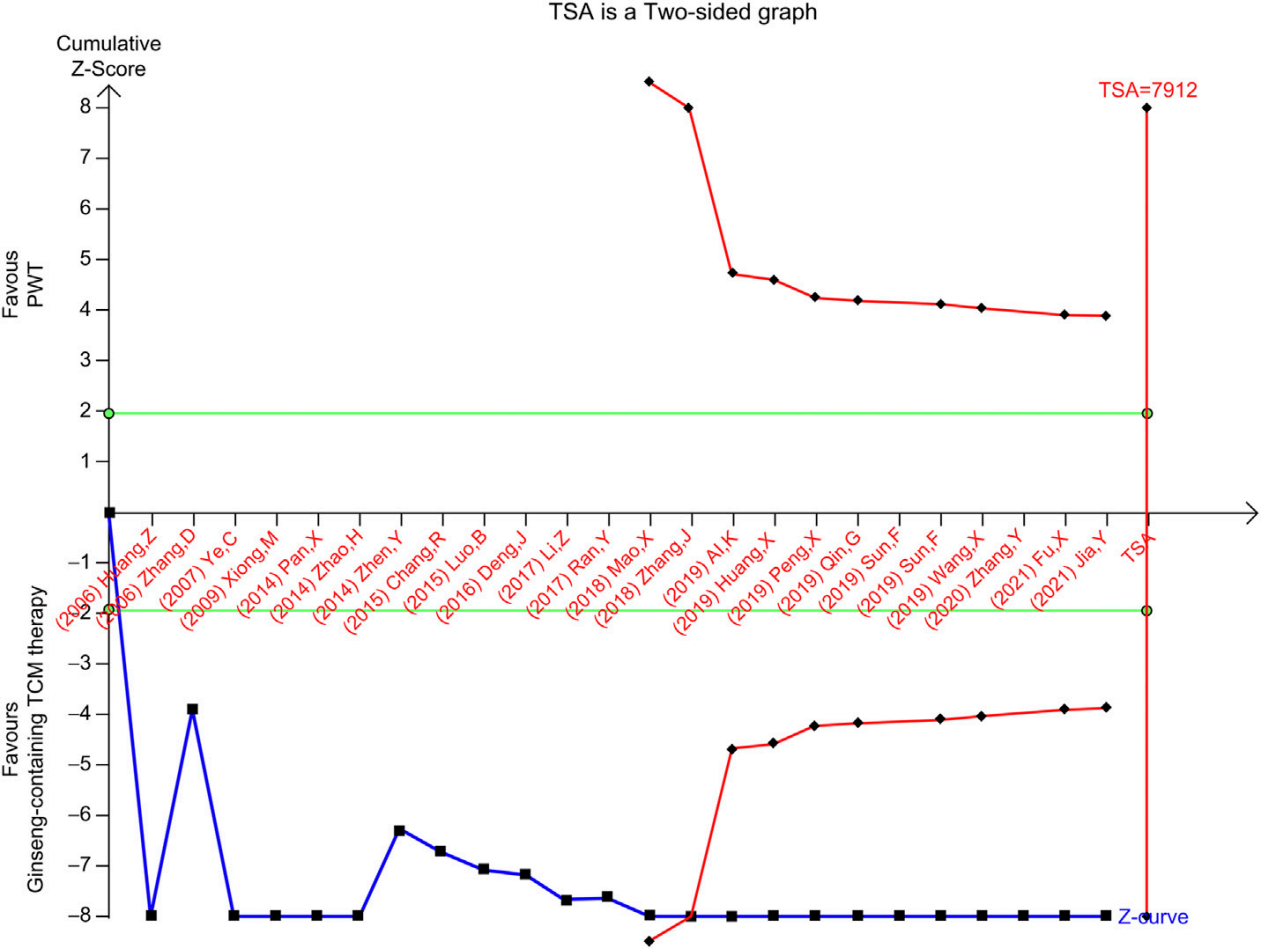
TSA of LVEF, left ventricular ejection fraction; TSA, trial sequential analysis.

In contrast, the RIS for the TSA of LVEF was 7,912 participants based on the intervention effect. In the meta-analysis of the effect of ginseng-containing TCM on LVEF, given by the empirical bias trials, the point estimate of the possible intervention effect was an MD of 5.97. The cumulative Z-curve (blue line) passes the conventional statistical boundary (green line) and the trial sequential monitoring boundary for benefit (red inward sloping line) but not the RIS boundary (red line), indicating the availability of sufficient evidence to prove that ginseng-containing TCM may enhance LVEF; however, the expected values were not attained (Figure 6).

### 3.10 Publication bias

Publication bias was assessed on the outcomes of more than 10 trials. Egger’s test was conducted to confirm publication bias. The results showed that both the LVEF and HFES were reliable (LVEF, *p* = 0.4881, HFES, *p* = 0.1037).

## 4 Discussion

Our meta-analysis first evaluated the clinical efficacy of ginseng-containing TCM in patients with ADHF with 28 studies. The results indicated that ginseng-containing TCM had a superior clinical efficacy, and improved ADHF patients’ LVEF, heart function with less adverse reactions and cases. Subgroup analysis indicated that ginseng preparations improved LVEF and clinical outcomes in heart failure patients regardless of the drug type, treatment duration and the patient’s LVEF.

### 4.1 Possible mechanism for ginseng-containing TCM

**FIGURE 7:**
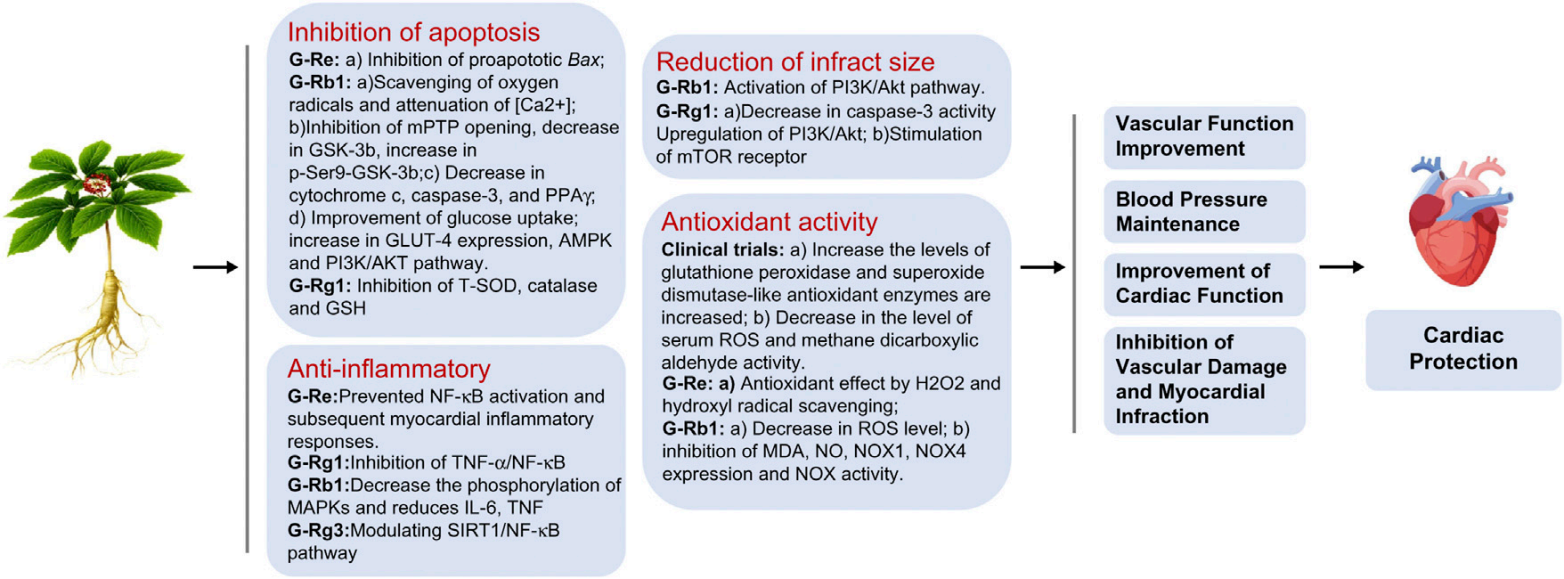
Ginseng’s cardiac protection mechanism.

Chinese medicine, especially ginseng-containing TCM, plays an indispensable role in the integrated Chinese and Western medicine treatment of cardiovascular diseases in China and is widely used in patients with ADHF. Ginsenosides are the most active ingredients of ginseng. More than 300 ginsenosides have been isolated, and the most widely studied include Rg1, Rb1, Re, and Rg3. Available studies have demonstrated that ginsenosides exert their anti-heart failure effects mainly through the inhibition of apoptosis, anti-inflammatory response (Ma et al., 2014; Chen R. C. et al., 2016; Tang et al., 2016; Ren et al., 2021), antioxidant activity (Hyun et al., 2022), and reduction of infarct size (Kim, 2018) (Figure 7). In TCM, qi deficiency is the main pathogenic mechanism underlying heart failure, and ginseng tonifies vital energy to restore yang and rescue rebellion. However, evidence of the efficacy of ginseng-containing TCM for treating ADHF is limited.

### 4.2 Ginseng-containing TCM improve ADHF patients’ clinical efficacy, LVEF, heart function with less adverse reactions and cases

The main outcomes of our meta-analysis are clinical efficacy and LVEF. HFES and CSS were two mostly used measurements in the including study. HFES was defined with the NYHA improvement while CSS was defined by the change in Chinese syndrome. It is worth pointing out that in most of the studies, patients with ADHF were rarely treated according to syndrome differentiation, which is contrary to the theory of Chinese medicine. Only four studies mentioned TCM syndrome, including Qi and Yin deficiency, Heart and Kidney Yang deficiency, Yang and Qi deficiency and Yang deficiency and Blood stasis. This is one of the reasons why CSS has not been reported in most studies. LVEF is another cornerstone of HF diagnosis, characterization, prognosis, patient triage and treatment selection. In our study, we find that with ginseng-containing TCM, the LVEF of patients could be improved.

Left ventricular remodeling is an important etiology of heart failure. Several studies have discovered that LVEDD, LVEDV, and SV are important prognostic predictors in patients with cardiovascular disease (recurrence of atrial fibrillation during hospitalization) (Solomon et al., 2010; Kajimoto et al., 2018). The results of this study suggest that ginseng-containing TCM treatments can reduce LVEDD and LVEDV and improve SV in patients, implying that ginseng may inhibit left ventricular remodeling in ADHF. However, limited data may lead to false-positive outcomes, and the high heterogeneity may be due to differences in patients at baseline (weight, gender and WT). Therefore, further studies should be performed to confirm its efficacy.

In recent studies, BNP and NT-proBNP levels were considered independent predictors of mortality in patients with ADHF. Higher BNP or NT-proBNP levels at admission have been reported to predict mortality risk in patients (Santaguida et al., 2014; Schaub et al., 2015). In our study, ginseng-containing TCM led to a reduction in BNP and NT-proBNP levels in patients with ADHF. However, none of included studies reported a half-year or 1-year survival rate. Therefore, we did not know whether ginseng-containing TCM improved survival rates.

Poor quality of life is another problem in patients with ADHF. Approximately 50% of older hospitalized patients with ADHF were frail, and 48% were pre-frail in this study. A 6-MWT limited to 150 m is common in patients with ADHF (Pandey et al., 2019). In our study, the 6-MWT improved and MLHFQ scores reduced, following treatment with ginseng-containing TCM.

Most of the time, ginseng has been reported to protect heart tissues from damage (Davis and Behm, 2019); however, cardiotoxic effects have also been recorded in some clinical and experimental publications (Parlakpinar et al., 2019). Our study also listed adverse reactions and cases in trials conducted on the safety of ginseng-containing TCM in patients with ADHF. Ten trials reported adverse cases out of the 17 RCTs that recorded adverse reactions. According to the report, dizziness, renal dysfunction, nausea, and vomiting are the most common adverse reactions and cases during the treatment of ADHF. Moreover, ginseng-containing TCM treatment did not increase the risk of adverse reactions and cases; instead, it reduced dizziness, suggesting the safety of ginseng-containing TCM.

### 4.3 Comparison with other related study

To our knowledge, only one meta-analysis of botanical drugs interventions in ADHF has been conducted in the literature (Ji, 2019). Compare with the meta-analysis conducted by Ji et al., which included nine RCTs, our study improved the search strategy by including 28 studies containing ginseng, confirming the clinical efficacy of Chinese botanical drugs containing ginseng for patients with ADHF. In addition, TSA was performed to increase the credibility of the results. The addition of 6-month readmission rate, BNP, MLHFQ, and specific analysis of side effects or outcomes provided a complete theoretical basis for the efficacy and safety of ginseng-containing TCM in the treatment of ADHF.

### 4.4 Source of heterogeneity

Since most results are of a high heterogeneity, we performed a subgroup analysis according to drug type, treatment duration and LVEF values. The results of the subgroup analysis were consistent with the overall study results indicating Ginseng-containing TCM is effective in patients with ADHF, independent of drug types, treatment period and patient EF value. For some indicators, heterogeneity decreased after subgroup analysis. When interacted with only SFI (before, I^2:^89%, SFI, I^2^:42%) or SGMI (before, I^2^:89%, SGMI, I^2^:0%) or QLQX (before, I^2^:89%, QLQX I^2^:0%), heterogeneity of LVEF decreased. For NT-proBNP, after subgroup analysis by EF value, heterogeneity decreased (before, I^2^:89%, EF ≤ 50, I^2^:0%; EF not limited, I^2^:0%). The above analysis suggests that drug types, LVEF values and treatment duration may be a source of heterogeneity.

Another reason for the heterogeneity was the uncertain risk of bias and low quality of the included studies. According to the Rob two measurement, twenty-five studies were with some concerns because of without mentioning blinding methods and allocation hiding. The quality of the included studies was measured by the modified Jadad Scores and. Results suggesting 14 included studies are of low quality. One study was measured with a high risk and the Jadad score was 1, which can also brought the heterogeneity. Lastly, the complexity of ADHF, etiology, disease history, nursing treatment, western treatment strategies, and ginseng origin may all contribute to heterogeneity.

### 4.5 Insights from the latest guideline

Latest, the 2022 AHA/ACC/HFSA Guideline for the Management of Heart failure was published. There are some differences from the earlier guideline. Firstly, the classification of HF based on LVEF was revised. Heart failure with improved ejection fraction (HFimpEF) was invented in the guideline. The HFimpEF was defined as previous LVEF ≤40% and a follow-up measurement of LVEF >40%. Secondly, SGLT-2i was recommended to HFrEF to reduce the rehospitalization. The above changes in the new guidelines imply us that we can explore ginseng-containing TCM in HFimpEF as well as the RCT interacted with the combine of ginseng and SGLT-2i.

### 4.6 Limitations

This study had several limitations. Firstly, a high risk of bias exists owing to the lack of blinding and unclear randomization methods. The low quality of the included studies could weaken the confidence of the results. Secondly, substantial heterogeneity was observed in most outcomes, except for efficacy and adverse reaction and cases. Additionally, oral take ginseng-related decoction was less reported in the ADHF clinical trials. Due to patent protection, we are only able to list the main components of the drugs included, but the extraction process and dosage of the botanical drugs are not known. None of the study reported the quality control and chemical analysis. Finally, few reports of follow-up and re-hospitalization recorded in RCTs, leading to the failure of our study to analyses ginseng on the survival of ADHF patients.

### 4.7 Future directions

All in all, this meta-analysis implies future directions for clinical trials as follow. Firstly, more high-quality RCTs should be conducted to strengthen this evidence, focusing on implementing subject-centered randomization, allocation concealment, and blinding. Moreover, RCTs should be reported completely and comprehensively using the consolidated standards of reporting trials statement (Schulz et al., 2010), with emphasis on the reporting of LVEF values, medical history, rehospitalization rates, and follow-up to identify sources of heterogeneity and clarify the prognosis of patients with ADHF. Thirdly, network meta-analysis can also be performed to compare the differences in ginseng-containing TCM use. More research in specific areas is required to assess the role of these factors in heterogeneity fully, and more clinical trials interacted by oral ginseng-related decoction could be performed to enhance the evidence for ginseng-containing TCM in ADHF. Lastly, with the knowledge of ADHF developed, we can also explore ginseng-containing TCM in HFimpEF as well as the RCT interacted with the combine of ginseng and SGLT-2i.

## 5 Conclusion

Summarily, compared with WT alone, ginseng-containing TCM is a possible way to benefit ADHF patients. However, the risk of bias and the studies’ quality reduced the credibility of the results. Therefore, we should pay more attention to ginseng-containing TCM clinical trials on patients with ADHF. More carefully designed larger-sample, long-term follow-up RCTs should be conducted in the future to provide reliable evidence for the use of ginseng-containing TCM in treating ADHF.

## Data Availability

The original contributions presented in the study are included in the article/supplementary material, further inquiries can be directed to the corresponding author.

## Abbreviations

ADHF, Acute decompensated heart failure; BNP, Brain natriuretic peptide; CI, Confidence interval; CSS, Chinese syndrome score; HFES, Heart function efficiency standard; HFimpEF, Heart failure with improved ejection fraction; LVEF, Left ventricular ejection fraction; LVEDD, Left ventricular end-diastolic diameter; LVEDV, Left ventricular end-diastolic volume; MD, Mean difference; MLHFQ, Minnesota living heart failure questionnaire; NT-proBNP, N-terminal pro-B-type natriuretic peptide; QLXX, Qili Qiangxin; RIS, Required information size; RCTs, Randomized controlled trials; RR, Risk ratio; REM, random-effects model; SV, Stroke volume; SMI, Shenmai injection; SGMI, Shengmai injection; SFI, Shenfu injection; 6-MWT, Six-minute walk test; TCM, Traditional Chinese medicine; TSA, Trial sequential analysis; WT, Western therapy; YXST, Yangxinshi tablets.
