# Supplementary material for: The efficacy of ginseng-containing traditional Chinese medicine in patients with acute decompensated heart failure: A systematic review and meta-analysis
Source: Front Pharmacol. 2023 Jan 10;13:1083001. doi: 10.3389/fphar.2022.1083001 (PMC9871309; doi:10.3389/fphar.2022.1083001)
Supplement: Supplementary file 1 [file Table1.docx]

Supplementary Material

# Supplementary Data

**Search strategy on PubMed**

#1 Acute Heart Failure [Mesh] OR Acute Decompensated Heart Failure OR Acute Decompensated Cardiac failure OR Acute Decompensated Myocardial failure OR Heart Decompensation OR ADHF OR Ventricular Dysfunction

#2 Panax [Mesh] OR panax* OR ginseng* OR eleuthero coccus OR jen shen* OR schinseng* OR ninjin* OR renshen* OR ren seng OR shen* jen OR insam OR hong shen OR ginsana

#3 shengmai* OR Yangxinshi OR Yixinshu OR Qili Qiangxin OR Shen* OR Shexiang Baoxin OR YiQi fumai OR zhenyuan OR Ginseng glycopeptide OR Tongxinluo

#4 #1 AND (#2 OR #3)

**Search strategy on Embase**

#1(Acute Heart Failure) OR (('Acute Heart Failure')/exp) OR (Acute Decompensated Heart Failure) OR (Acute Decompensated Cardiac failure) OR (Acute Decompensated Myocardial failure) OR (Heart Decompensation) OR (ADHF) OR (Ventricular Dysfunction)

#2 (Panax)/exp OR (panax) OR (ginseng) OR (eleuthero coccus) OR (jen shen) OR (schinseng) OR (ninjin) OR (renshen) OR (ren seng) OR (‘shen jen’) OR (insam) OR (hong shen) OR (ginsana) OR (shengmai) OR (Yangxinshi) OR (Yixinshu) OR (Qili Qiangxin) OR (Shen*) OR (Shexiang Baoxin) OR (‘YiQi fumai’) OR (zhenyuan) OR (‘Ginseng glycopeptide’) OR (Tongxinluo)

#3 #1 And #2

**Search strategy on Cochrane**

#1 Acute Heart Failure [Mesh] OR (Acute Decompensated Heart Failure):ti,ab,kw OR (Acute Decompensated Cardiac failure):ti,ab,kw OR (Acute Decompensated Myocardial failure):ti,ab,kw OR (Heart Decompensation):ti,ab,kw OR (ADHF):ti,ab,kw OR (Ventricular Dysfunction):ti,ab,kw

#2 Panax [Mesh] OR (panax):ti,ab,kw OR (ginseng):ti,ab,kw OR (eleuthero coccus):ti,ab,kw OR (jen shen):ti,ab,kw OR (schinseng):ti,ab,kw OR (ninjin):ti,ab,kw OR (renshen):ti,ab,kw OR (ren seng):ti,ab,kw OR (shen jen):ti,ab,kw OR (insam):ti,ab,kw OR (hong shen):ti,ab,kw OR (ginsana):ti,ab,kw

#3 (shengmai):ti,ab,kw OR (Yangxinshi):ti,ab,kw OR (Yixinshu):ti,ab,kw OR (Qili Qiangxin):ti,ab,kw OR (Shexiang Baoxin):ti,ab,kw OR (YiQi fumai):ti,ab,kw OR (zhenyuan):ti,ab,kw OR (Ginseng glycopeptide):ti,ab,kw OR (Tongxinluo):ti,ab,kw

#1 AND (#2 OR #3)

**Search strategy on CNKI**

( ( ( ( ( ( ( ( ( ( ( 主题%='参' or 题名%='参' ) OR ( 主题%='生脉' or 题名%='生脉' ) ) OR ( 主题%='养心氏' or 题名%='养心氏' ) ) OR ( 主题%='益心舒' or 题名%='益心舒' ) ) OR ( 主题%='芪苈强心' or 题名%='芪苈强心' ) ) OR ( 主题%='麝香保心' or 题名%='麝香保心' ) ) OR ( 主题%='益气复脉' or 题名%='益气复脉' ) ) OR ( 主题%='振源' or 题名%='振源' ) ) OR ( 主题%='通心络' or 题名%='通心络' ) ) ) AND ( ( ( ( 主题%='慢性心力衰竭急性发作' or 题名%='慢性心力衰竭急性发作' ) OR ( 主题%='慢性心力衰竭失代偿' or 题名%='慢性心力衰竭失代偿' ) ) )

**Search strategy on Wanfang**

(主题:(人参) or 主题:(生脉) or 主题:(益气复脉) or 主题:(养心氏) or 主题:(益心舒) or 主题:(芪苈强心) or 主题:(麝香保心)or 主题:(通心络) or 主题:(振源)or 主题:(参)) and (主题:(慢性心力衰竭失代偿) or 主题:(慢性心力衰竭急性发作))

**Search strategy on Sinomed**

("慢性心力衰竭急性发作"[常用字段:智能] OR "失代偿性心力衰竭"[常用字段:智能] OR "急性心力衰竭"[常用字段:智能] OR "急性心衰"[常用字段:智能]) AND ("参"[常用字段:智能] OR "麝香保心"[常用字段:智能] OR "芪苈强心"[常用字段:智能] OR "生脉"[常用字段:智能] OR "养心氏"[常用字段:智能] OR "益气复脉"[常用字段:智能] OR "振源"[常用字段:智能] OR "通心络"[常用字段:智能])

**Search strategy on VIP**

(M=参 OR M=生脉 OR M=益气复脉 OR M=养心氏 OR M=益心舒 OR M=芪苈强心 OR M=麝香保心 OR M=益气复脉 OR M=振源 OR M=通心络) AND (M=慢性心力衰竭急性发作 OR M=慢性心衰急性发作 OR M=心衰失代偿 OR M=心衰急性发作 OR M=慢性心力衰竭失代偿 OR M=急性心力衰竭 OR M=急性心衰)

# Supplementary Figures and Tables

## Supplementary Figures


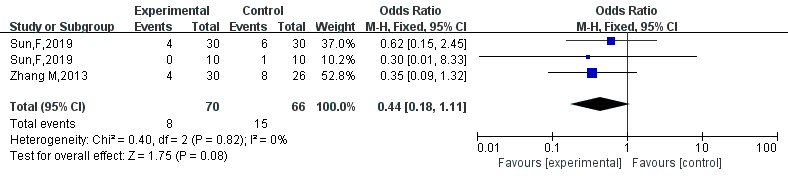


**Supplementary Figure 1 Meta-analysis of rehospitalization between two groups**


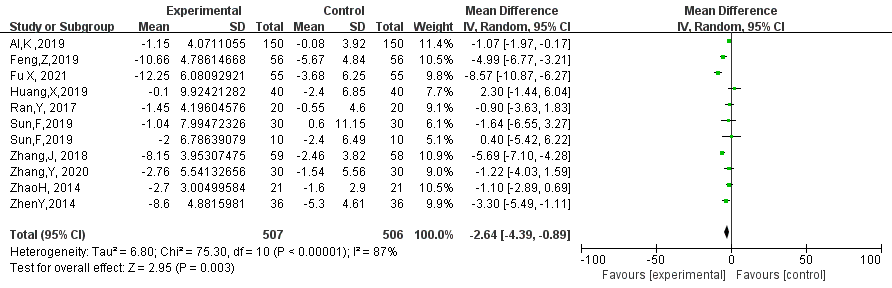


**Supplementary Figure 2 Meta-analysis of LVEDD between two groups**


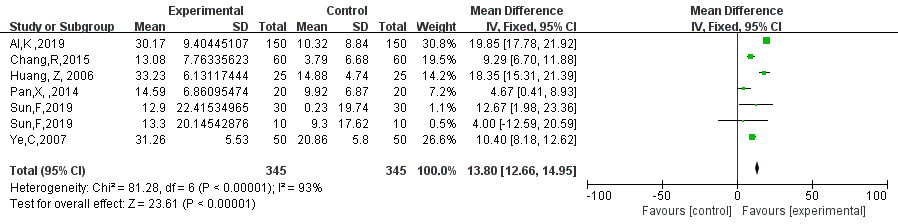


**Supplementary Figure 3 Meta-analysis of SV between two groups**


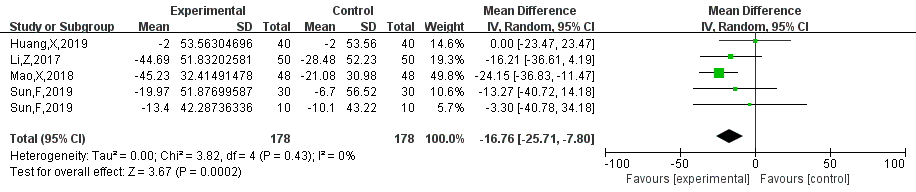


**Supplementary Figure 4 Meta-analysis of LVEDV between two groups**


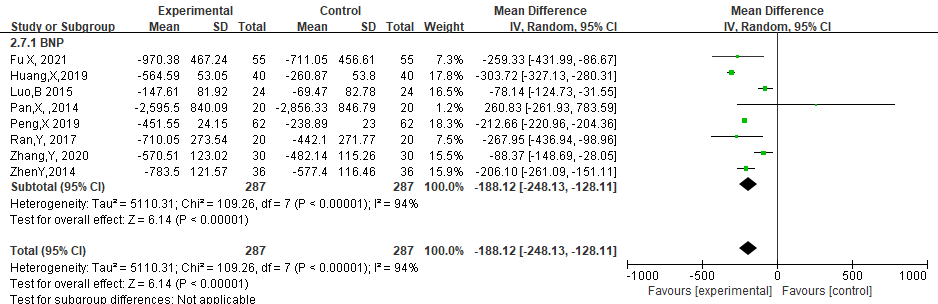


**Supplementary Figure 5 Meta-analysis of BNP between two groups**

r
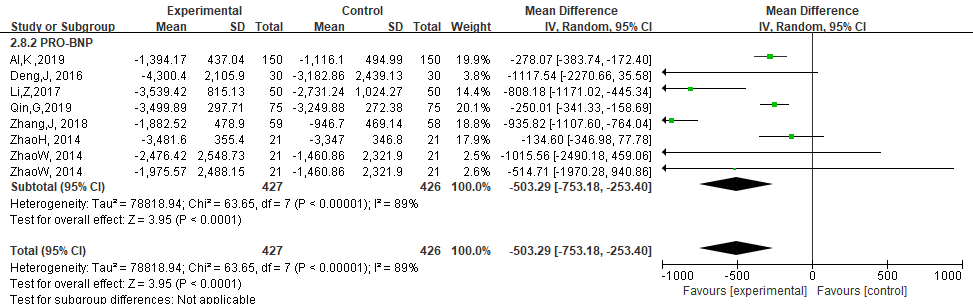


**Supplementary Figure 6: Meta-analysis of NT-proBNP between two groups**


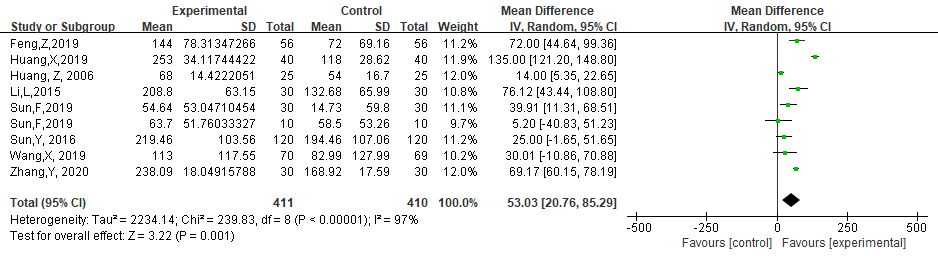


**Supplementary Figure 7: Meta-analysis of 6-MWT between two groups**


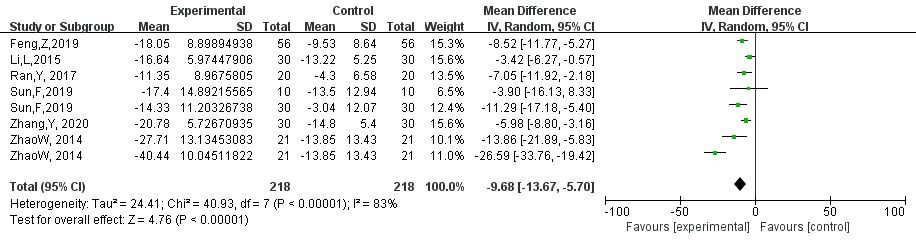


**Supplementary Figure 8: Meta-analysis of MLFHQ between two groups**


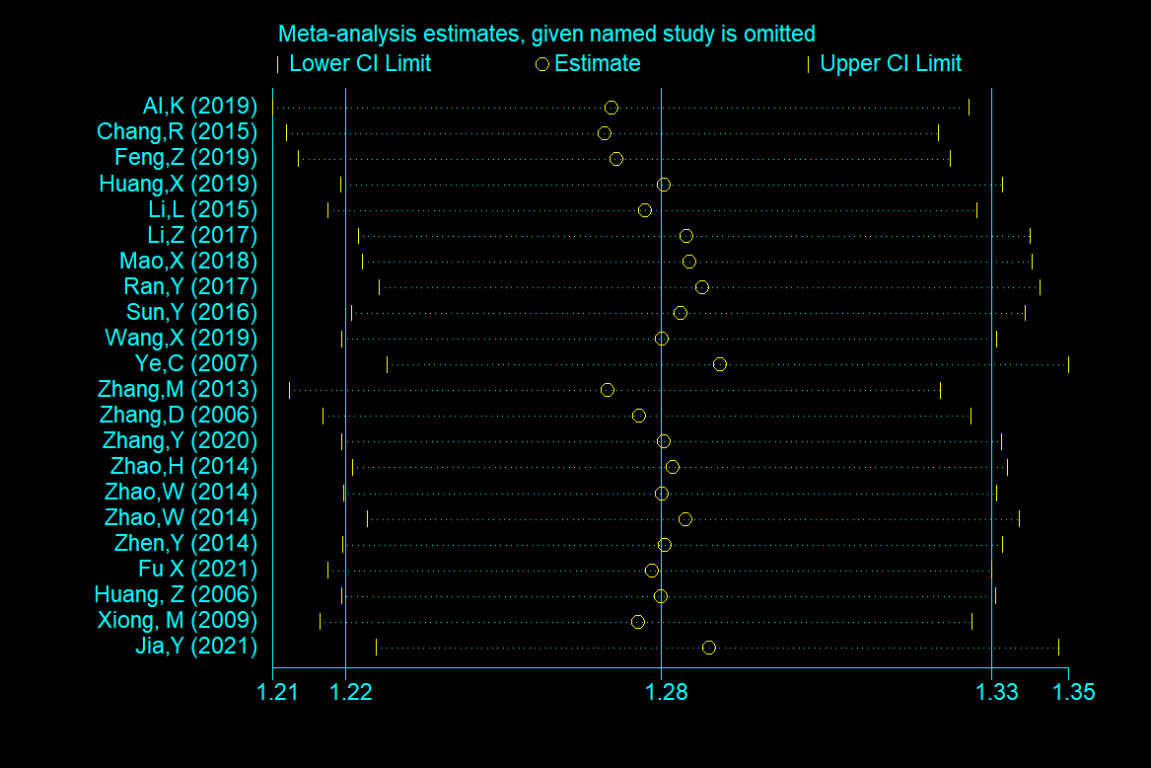


**Supplementary Figure 9: Sensitivity analysis of HFES between two groups**


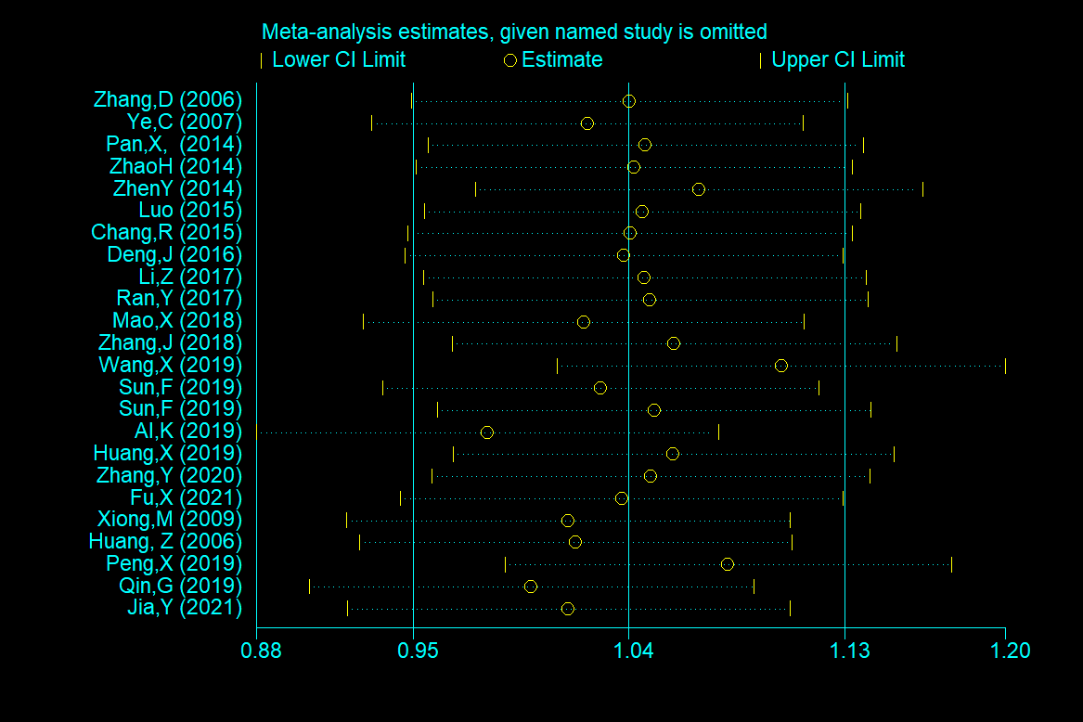


**Supplementary Figure 10: Sensitivity analysis of LVEF between two groups**

## Supplementary Tables

**Supplementary Table 1: PRISMA2020 checklist**

| **Section and Topic** | **Item #** | **Checklist item** | **Location where item is reported** |
| --- | --- | --- | --- |
| **TITLE** | | |  |
| Title | 1 | Identify the report as a systematic review. | Page 1 |
| **ABSTRACT** | | |  |
| Abstract | 2 | See the PRISMA 2020 for Abstracts checklist. | Line14 to line 32 |
| **INTRODUCTION** | | |  |
| Rationale | 3 | Describe the rationale for the review in the context of existing knowledge. | Line44 to line63 |
| Objectives | 4 | Provide an explicit statement of the objective(s) or question(s) the review addresses. | Line60 to line63 |
| **METHODS** | | |  |
| Eligibility criteria | 5 | Specify the inclusion and exclusion criteria for the review and how studies were grouped for the syntheses. | Line 81 to line104 |
| Information sources | 6 | Specify all databases, registers, websites, organisations, reference lists and other sources searched or consulted to identify studies. Specify the date when each source was last searched or consulted. | Line 73 |
| Search strategy | 7 | Present the full search strategies for all databases, registers and websites, including any filters and limits used. | Line 70 to 80 |
| Selection process | 8 | Specify the methods used to decide whether a study met the inclusion criteria of the review, including how many reviewers screened each record and each report retrieved, whether they worked independently, and if applicable, details of automation tools used in the process. | Line 105 to 111 |
| Data collection process | 9 | Specify the methods used to collect data from reports, including how many reviewers collected data from each report, whether they worked independently, any processes for obtaining or confirming data from study investigators, and if applicable, details of automation tools used in the process. | Line 105 to 111 |
| Data items | 10a | List and define all outcomes for which data were sought. Specify whether all results that were compatible with each outcome domain in each study were sought (e.g. for all measures, time points, analyses), and if not, the methods used to decide which results to collect. | Line 123 to line 136 |
|  | 10b | List and define all other variables for which data were sought (e.g. participant and intervention characteristics, funding sources). Describe any assumptions made about any missing or unclear information. | Line 123 to line 136 |
| Study risk of bias assessment | 11 | Specify the methods used to assess risk of bias in the included studies, including details of the tool(s) used, how many reviewers assessed each study and whether they worked independently, and if applicable, details of automation tools used in the process. | Page 6 |
| Effect measures | 12 | Specify for each outcome the effect measure(s) (e.g. risk ratio, mean difference) used in the synthesis or presentation of results. | Page 7 |
| Synthesis methods | 13a | Describe the processes used to decide which studies were eligible for each synthesis (e.g. tabulating the study intervention characteristics and comparing against the planned groups for each synthesis (item #5)). | Page 5,6 |
|  | 13b | Describe any methods required to prepare the data for presentation or synthesis, such as handling of missing summary statistics, or data conversions. | Page 5,6 |
|  | 13c | Describe any methods used to tabulate or visually display results of individual studies and syntheses. | Page 5,6 |
|  | 13d | Describe any methods used to synthesize results and provide a rationale for the choice(s). If meta-analysis was performed, describe the model(s), method(s) to identify the presence and extent of statistical heterogeneity, and software package(s) used. | Line112 to line122 |
|  | 13e | Describe any methods used to explore possible causes of heterogeneity among study results (e.g. subgroup analysis, meta-regression). | Line146 to line 150 |
|  | 13f | Describe any sensitivity analyses conducted to assess robustness of the synthesized results. | Line 144 to 245 |
| Reporting bias assessment | 14 | Describe any methods used to assess risk of bias due to missing results in a synthesis (arising from reporting biases). | Line 117 to 122 |
| Certainty assessment | 15 | Describe any methods used to assess certainty (or confidence) in the body of evidence for an outcome. | Line 137 |
| **RESULTS** | | |  |
| Study selection | 16a | Describe the results of the search and selection process, from the number of records identified in the search to the number of studies included in the review, ideally using a flow diagram. | Flow chart (fig 1) |
|  | 16b | Cite studies that might appear to meet the inclusion criteria, but which were excluded, and explain why they were excluded. | Flow chart (fig 1) |
| Study characteristics | 17 | Cite each included study and present its characteristics. | Line 166  Table1 |
| Risk of bias in studies | 18 | Present assessments of risk of bias for each included study. | Fig2, S5 |
| Results of individual studies | 19 | For all outcomes, present, for each study: (a) summary statistics for each group (where appropriate) and (b) an effect estimate and its precision (e.g. confidence/credible interval), ideally using structured tables or plots. | Line199 to line280  Fig3,4, Supplementary figureS1 to figure S8 |
| Results of syntheses | 20a | For each synthesis, briefly summarise the characteristics and risk of bias among contributing studies. | Line199 to line280  Fig3,4, Supplementary figureS1 to figure S8 |
|  | 20b | Present results of all statistical syntheses conducted. If meta-analysis was done, present for each the summary estimate and its precision (e.g. confidence/credible interval) and measures of statistical heterogeneity. If comparing groups, describe the direction of the effect. | Line199 to line280  Fig3,4, Supplementary figureS1 to figure S8 |
|  | 20c | Present results of all investigations of possible causes of heterogeneity among study results. | Line199 to line280  Fig3,4, Supplementary figureS1 to figure S8 |
|  | 20d | Present results of all sensitivity analyses conducted to assess the robustness of the synthesized results. | Line 274 to line306 |
| Reporting biases | 21 | Present assessments of risk of bias due to missing results (arising from reporting biases) for each synthesis assessed. | Line 274 to line306 |
| Certainty of evidence | 22 | Present assessments of certainty (or confidence) in the body of evidence for each outcome assessed. | Line 274 to line306 |
| **DISCUSSION** | | |  |
| Discussion | 23a | Provide a general interpretation of the results in the context of other evidence. | Line 324 to line 331 |
|  | 23b | Discuss any limitations of the evidence included in the review. | Line 367 to line 374 |
|  | 23c | Discuss any limitations of the review processes used. | Line 367 to line 374 |
|  | 23d | Discuss implications of the results for practice, policy, and future research. | Line 382 |
| **OTHER INFORMATION** | | |  |
| Registration and protocol | 24a | Provide registration information for the review, including register name and registration number, or state that the review was not registered. | Line 73 |
|  | 24b | Indicate where the review protocol can be accessed, or state that a protocol was not prepared. | Line 73 |
|  | 24c | Describe and explain any amendments to information provided at registration or in the protocol. | Line 73 |
| Support | 25 | Describe sources of financial or non-financial support for the review, and the role of the funders or sponsors in the review. | Line 396 |
| Competing interests | 26 | Declare any competing interests of review authors. | Line 388 |
| Availability of data, code and other materials | 27 | Report which of the following are publicly available and where they can be found: template data collection forms; data extracted from included studies; data used for all analyses; analytic code; any other materials used in the review. | Line 73 |

*From:*  Page MJ, McKenzie JE, Bossuyt PM, Boutron I, Hoffmann TC, Mulrow CD, et al. The PRISMA 2020 statement: an updated guideline for reporting systematic reviews. BMJ 2021;372:n71. doi: 10.1136/bmj.n71

For more information, visit: http://www.prisma-statement.org/

**Supplementary Table 2 Ginseng-containing TCM included in the study**

| Intervention | Search Terms | References |
| --- | --- | --- |
| Ginseng-containing TCM | Panax [Mesh] OR panax* OR ginseng* OR eleuthero coccus OR jen shen* OR schinseng* OR ninjin* OR renshen* OR ren seng OR shen* jen OR insam OR hong shen OR ginsana | (1, 2) |
| Ginseng-containing injection | Shenfu OR shenmai OR shengmai OR YiQi fumai OR Ginseng glycopeptide | (3, 4) |
| Ginseng- containing Prepared oral Chinese Medicine | Dengzhan shengmai OR Yangxinshi OR Yixinshu OR Qili Qiangxin OR Shensong Yangxin OR Shexiang Baoxin OR Shenfu Qiangxin OR Guanxin shengmai OR Tongxinluo | (5-7) |

**Supplementary Table 3 Ongoing clinical trials (search date 2022.07.10)**

| **Registration number** | **Public title** | **Primary sponsor** | **Study type** | **Register date** |
| --- | --- | --- | --- | --- |
| ChiCTR-TRC-12002857 | Shenfu injection in the treatment of Coronary heart disease acute exacerbation of chronic heart failure (yangqi kuixu zheng), a multicenter randomized, blind, controlled trial | The First Affiliated HospitaI of Tianjin University of TraditionaI Chinese Medicine | Interventional studies | 2012/12/25 |
| ChiCTR1900026086 | Effect of Shen-Fu injection on hemodynamics and clinical outcomes in patients with cardiogenic shock in acute decompensated heart failure: a multicenter, stratified-randomized, patient and assessor-blinded, single-dummy, placebo-controlled trial | The First Affiliated Hospital of Guangzhou University of Chinese Medicine | Interventional studies | 2019/09/20 |

**Supplementary Table 4 Rob2 assessment for included trials**

|  | Domain 1. Randomization process | | | | | | Domain 2. Deviations from intended interventions | | | | | Domain 3. Mising outcome data | | | | | Domain 4. Measurement of the outcome | | | | | | | | Domain 5. Selection of the reported result | | | | Overall | |
| --- | --- | --- | --- | --- | --- | --- | --- | --- | --- | --- | --- | --- | --- | --- | --- | --- | --- | --- | --- | --- | --- | --- | --- | --- | --- | --- | --- | --- | --- | --- |
| Study ID | 1 | 2 | Note for 1&2 | 3 | Note for 3 | result | 1-2 | Note for 1&2 | 5-6 | result | 1 | | Note for 1 | 2-4 | result | 1 | | Note for 1 | 2 | 3 | 4 | 5 | result | 1 | | 2-3 | result | overall | |  |
| Huang, Z | PY | PY | * | N | Described. | Low | NI | Unclear | Y | SC | Y | | complete. | NA | Low | N | | Complete. | N | PY | PN | NA | Low | Y | | N | Low | SC | |  |
| Zhang, D | Y | PY | Randomized Table | N | Described. | Low | NI | Unclear | Y | SC | Y | | complete. | NA | Low | N | | Complete. | N | PY | PN | NA | Low | Y | | N | Low | SC | |  |
| Ye, C | PY | PY | * | N | Described. | Low | NI | Unclear | Y | SC | Y | | complete. | NA | Low | N | | Complete. | N | PY | PN | NA | Low | Y | | N | Low | SC | |  |
| Xiong, M | PY | PY | * |  | Described. | Low | NI | Unclear | Y | SC | Y | | complete. | NA | Low | N | | Complete. | N | PY | PN | NA | Low | Y | | N | Low | SC | |  |
| Zhang, M | Y | PY | Randomized Table | N | Described. | Low | NI | Unclear | Y | SC | Y | | complete. | NA | Low | N | | Complete. | N | PY | PN | NA | Low | Y | | N | Low | SC | |  |
| Pan, X | PY | PY | * | N | Described. | Low | NI | Unclear | Y | SC | Y | | complete. | NA | Low | N | | Complete. | N | PY | PN | NA | Low | Y | | N | Low | SC | |  |
| Zhao, H | PY | PY | * | N | Described. | Low | NI | Unclear | Y | SC | Y | | complete. | NA | Low | N | | Complete. | N | PY | PN | NA | Low | Y | | N | Low | SC | |  |
| Zhao, W | Y | PY | Randomized Table | N | Described. | Low | NI | Unclear | Y | SC | Y | | complete. | NA | Low | N | | Complete. | N | PY | PN | NA | Low | Y | | N | Low | SC | |  |
| Zhen,Y | Y | PY | Randomized Table | N | Described. | Low | NI | Unclear | Y | SC | Y | | complete. | NA | Low | N | | Complete. | N | PY | PN | NA | Low | Y | | N | Low | SC | |  |
| Li, L | Y | PY | Randomized Table | N | Described. | Low | NI | Unclear | Y | SC | Y | | complete. | NA | Low | N | | Complete. | N | PY | PN | NA | Low | Y | | N | Low | SC | |  |
| Luo, B | PY | PY | * | N | Described. | Low | NI | Unclear | Y | SC | Y | | complete. | NA | Low | N | | Complete. | N | PY | PN | NA | Low | Y | | N | Low | SC | |  |
| Chang, R | PY | PY | * | N | Described. | Low | NI | Unclear | Y | SC | Y | | complete. | NA | Low | N | | Complete. | N | PY | PN | NA | Low | Y | | N | Low | SC | |  |
| Deng, J | PY | PY | * | N | Described. | Low | N | Placebo were used | N | Low | Y | | complete. | NA | Low | N | | Complete. | N | PY | PN | NA | Low | Y | | N | Low | Low | |  |
| Sun, Y | PY | PY | * | N | Described. | Low | NI | Unclear | Y | SC | Y | | complete. | NA | Low | N | | Complete. | N | PY | PN | NA | Low | Y | | N | Low | SC | |  |
| Li, Z | Y | PY | Randomized Table | N | Described. | Low | NI | Unclear | Y | SC | Y | | complete. | NA | Low | N | | Complete. | N | PY | PN | NA | Low | Y | | N | Low | SC | |  |
| Ran, Y | PY | PY | * | N | Described. | Low | NI | Unclear | Y | SC | Y | | complete. | NA | Low | N | | Complete. | N | PY | PN | NA | Low | Y | | N | Low | SC | |  |
| Mao, X | PY | PY | * | N | Described. | Low | NI | Unclear | Y | SC | Y | | complete. | NA | Low | N | | Complete. | N | PY | PN | NA | Low | Y | | N | Low | SC | |  |
| Zhang, J | Y | PY | Randomized Table | N | Described. | Low | NI | Unclear | Y | SC | Y | | complete. | NA | Low | N | | Complete. | N | PY | PN | NA | Low | Y | | N | Low | SC | |  |
| Wang, X | Y | PY | Program random | N | Described. | Low | N | ** | N | Low | Y | | complete. | NA | Low | N | | Complete. | N | N | PN | NA | Low | Y | | N | Low | Low | |  |
| Feng, Z | PY | PY | * | N | Described. | Low | NI | Unclear | Y | SC | Y | | complete. | NA | Low | N | | Complete. | N | PY | PN | NA | Low | Y | | N | Low | SC | |  |
| Sun, F | PY | PN | Time Sequence | N | Described. | High |  | Unclear |  | SC | Y | | complete. | NA | Low | N | | Complete. | N | Y | PN | NA | Low | Y | | N | Low | High | |  |
| AI, K | Y | PY | Randomized Table | N | Described. | Low | NI | Unclear | Y | SC | Y | | complete. | NA | Low | N | | Complete. | N | PY | PN | NA | Low | Y | | N | Low | SC | |  |
| Huang, X | PY | PY | * | N | Described. | Low | NI | Unclear | Y | SC | Y | | complete. | NA | Low | N | | Complete. | N | PY | PN | NA | Low | Y | | N | Low | SC | |  |
| Zhang, Y | Y | PY | Randomized Table | N | Described. | Low | NI | Unclear | Y | SC | Y | | complete. | NA | Low | N | | Complete. | N | PY | PN | NA | Low | Y | | N | Low | SC | |  |
| Fu, X | Y | PY | Randomized Table was used. | N | Described. | Low | NI | Unclear | Y | SC | Y | | complete. | NA | Low | N | | Complete. | N | PY | PN | NA | Low | Y | | N | Low | SC | |  |
| Qin, G | PY | PY | * | N | Described. | Low | NI | Unclear | Y | SC | Y | | complete. | NA | Low | N | | Complete. | N | PY | PN | NA | Low | Y | | N | Low | SC | |  |
| Peng, X | Y | PY | Randomized Table was used. | N | Described. | Low | PY | Unclear | Y | SC | Y | | complete. | NA | Low | N | | Complete. | N | PY | PN | NA | Low | Y | | N | Low | SC | |  |
| Jia, Y | Y | PY | Randomized Table was used. | N | Described. | Low | PY | Unclear | Y | SC | Y | | complete. | NA | Low | N | | Complete. | N | PY | PN | NA | Low | Y | | N | Low | SC | |  |

Y: Yes.

PY: Probably yes.

N: No.

PN: Probably no.

SC: Some concerns.

*: Randomized was mentioned without randomized way

**: Placebo were used. The indicator and surveyor were blind.

**Supplementary Table 5 Jadad Scores for 28 studies**

|  | Randomization | Allocation hiding | Blinding | Withdrawais and Dropouts | Total |
| --- | --- | --- | --- | --- | --- |
| Huang, Z | 1 | 1 | 0 | 1 | 3 |
| Zhang, D | 1 | 1 | 0 | 1 | 3 |
| Ye, C | 1 | 1 | 0 | 1 | 3 |
| Xiong, M | 1 | 1 | 0 | 1 | 3 |
| Zhang, M | 2 | 1 | 0 | 1 | 4 |
| Pan, X | 1 | 1 | 0 | 1 | 3 |
| Zhao, H | 1 | 1 | 0 | 1 | 3 |
| Zhao, W | 2 | 1 | 0 | 1 | 4 |
| Zhen, Y | 2 | 1 | 0 | 1 | 4 |
| Li, L | 2 | 1 | 0 | 1 | 4 |
| Luo, B | 1 | 1 | 0 | 1 | 3 |
| Chang, R | 1 | 1 | 0 | 1 | 3 |
| Deng, J | 1 | 1 | 2 | 1 | 5 |
| Sun, Y | 1 | 1 | 0 | 1 | 3 |
| Li, Z | 2 | 1 | 0 | 1 | 4 |
| Ran, Y | 1 | 1 | 0 | 1 | 3 |
| Mao, X | 1 | 1 | 0 | 1 | 3 |
| Zhang, J | 2 | 1 | 0 | 1 | 4 |
| Wang, X | 2 | 2 | 2 | 1 | 7 |
| Feng, Z | 1 | 1 | 0 | 1 | 3 |
| Sun, F | 0 | 0 | 0 | 1 | 1 |
| AI, K | 2 | 1 | 0 | 1 | 4 |
| Huang, X | 1 | 1 | 0 | 1 | 3 |
| Zhang, Y | 2 | 1 | 0 | 1 | 4 |
| Fu, X | 2 | 1 | 0 | 1 | 4 |
| Qin, G | 1 | 1 | 0 | 1 | 3 |
| Peng, X | 2 | 1 | 0 | 1 | 4 |
| Jia, Y | 2 | 1 | 0 | 1 | 4 |

**Supplementary Table 6 Subgroup study of secondary outcomes**

| **Outcome or Subgroup** | **Studies** | **Participants** | **MD/RR (95% CI)** | **Z** | **P** | **Heterogeneity** | |
| --- | --- | --- | --- | --- | --- | --- | --- |
|  |  |  |  |  |  | **I^2^** | **P_h_** |
| 1.LVEDD | 10 | 1013 | -2.64 [-4.39, -0.89] | 2.95 | 0.003 | 87 | <0.00001 |
| different drug |  |  |  |  |  |  |  |
| SFI | 4 | 279 | -1.59 [-4.94, 1.75] | 0.93 | 0.35 | 89 | <0.00001 |
| Shenmai | 4 | 354 | -4.59 [-7.35, -1.82] | 3.25 | 0.001 | 84 | 0.0004 |
| YXS | 1 | 80 | -0.79 [-4.54, 2.96] | 0.41 | 0.68 | 0 | 0.6 |
| QLQX | 1 | 300 | -1.07 [-1.97, -0.17] | 2.32 | 0.02 | / | / |
| duration |  |  |  |  |  |  |  |
| <=14d | 8 | 633 | -3.14 [-5.21, -1.06] | 2.96 | 0.003 | 86 | <0.00001 |
| >14d | 2 | 380 | -1.05 [-1.93, -0.18] | 2.35 | 0.02 | 0 | 0.86 |
| LVEF |  |  |  |  |  |  |  |
| EF<=50% | 4 | 329 | -2.30 [-5.39, 0.78] | 1.46 | 0.14 | 85 | <0.00001 |
| EF not limited | 6 | 684 | -2.80 [-5.10, -0.51] | 2.39 | 0.02 | 87 | <0.00001 |
| 2.SV | 6 | 690 | 12.14 [7.33, 16.95] | 4.95 | <0.00001 | 92.8 | <0.00001 |
| different drug |  |  |  |  |  |  |  |
| Shengmai | 1 | 120 | 9.29 [6.70, 11.88] | 7.03 | <0.00001 | / | / |
| Shenmai | 3 | 190 | 11.26 [4.37, 18.15] | 3.2 | 0.001 | 93 | <0.00001 |
| YXS | 1 | 80 | 10.13 [1.14, 19.11] | 2.21 | 0.03 | 0 | 0.39 |
| QLQX | 1 | 300 | 19.85 [17.78, 21.92] | 18.84 | <0.00001 | / | / |
| duration |  |  |  |  |  |  |  |
| <=14d | 4 | 310 | 10.80 [6.01, 15.59] | 4.42 | <0.00001 | 91 | <0.00001 |
| >14d | 2 | 380 | 15.00 [6.63, 23.37] | 3.51 | 0.0004 | 60 | <0.00001 |
| LVEF |  |  |  |  |  |  |  |
| EF<=50% | 1 | 40 | 4.67 [0.41, 8.93] | 2.15 | 0.03 | / | / |
| EF not limited | 5 | 650 | 13.62 [8.72, 18.52] | 5.45 | <0.00001 | 92 | <0.00001 |
| 3.LVEDV | 4 | 356 | -16.76 [-25.71, -7.80] | 3.67 | 0.0002 | 0 | 0.43 |
| different drug |  |  |  |  |  |  |  |
| SFI | 3 | 276 | -16.27 [-29.52, -3.01] | 2.4 | 0.02 | 37 | 0.2 |
| YXS | 1 | 80 | -9.79 [-31.93, 12.36] | 0.87 | 0.39 | 0 | 0.67 |
| duration |  |  |  |  |  |  |  |
| <=14d | 3 | 276 | -16.27 [-29.52, -3.01] | 2.4 | 0.02 | 37 | 0.2 |
| >14d | 1 | 80 | -9.79 [-31.93, 12.36] | 0.87 | 0.39 | 0 | 0.67 |
| LVEF |  |  |  |  |  |  |  |
| EF<=50% | 2 | 180 | -9.19 [-24.93, 6.55] | 1.14 | 0.25 | 4 | 0.31 |
| EF not limited | 2 | 176 | -20.60 [-31.61, -9.60] | 3.67 | 0.0002 | 0 | 0.5 |
| 4.BNP | 8 | 574 | -181.50 [-295.16, -103.84] | 4.58 | <0.00001 | 98 | <0.00001 |
| different drug |  |  |  |  |  |  |  |
| SFI | 2 | 120 | -303.05 [-326.24, -279.85] | 25.61 | <0.00001 | 0 | 0.68 |
| Shenmai | 5 | 330 | -124.13 [-213.91, -34.35] | 2.71 | 0.007 | 88 | <0.00001 |
| Shengmai | 1 | 124 | -212.66 [-220.96, -204.36] | 50.21 | <0.00001 | / | / |
| duration |  |  |  |  |  |  |  |
| <=14d | 8 | 574 | -181.50 [-295.16, -103.84] | 4.58 | <0.00001 | 98 | <0.00001 |
| LVEF |  |  |  |  |  |  |  |
| EF<=50% | 5 | 300 | -137.63 [-283.86, 8.59] | 1.84 | 0.07 | 98 | <0.00001 |
| EF not limited | 3 | 274 | -212.90 [-221.18, -204.62] | 50.38 | <0.00001 | 0 | 0.71 |
| 5.NT-proBNP | 7 | 853 | -503.29 [-753.18, -253.40] | 3.95 | <0.0001 | 89 | <0.00001 |
| different drug |  |  |  |  |  |  |  |
| SFI | 5 | 403 | -691.80 [-1148.32, -235.28] | 2.97 | 0.003 | 86 | <0.00001 |
| QLQX | 1 | 300 | -278.07 [-383.74, -172.40] | 5.16 | <0.00001 | / | / |
| Shengmai | 1 | 150 | -250.01 [-341.33, -158.69] | 5.37 | <0.00001 | / | / |
| duration |  |  |  |  |  |  |  |
| <=14d | 6 | 553 | -584.30 [-940.73, -227.87] | 3.21 | 0.001 | 90 | <0.00001 |
| >14d | 1 | 300 | -278.07 [-383.74, -172.40] | 5.16 | <0.00001 | / | / |
| LVEF |  |  |  |  |  |  |  |
| EF<=50% | 3 | 277 | -916.10 [-1069.97, -762.23] | 11.67 | <0.00001 | 0 | 0.78 |
| EF not limited | 4 | 576 | -251.86 [-317.44, -186.29] | 7.53 | <0.00001 | 0 | 0.63 |
| 6.6MWT | 8 | 821 | 53.03 [20.76, 85.29] | 3.22 | 0.001 | 97 | <0.00001 |
| different drug |  |  |  |  |  |  |  |
| SFI | 3 | 459 | 64.43 [-19.54, 148.40] | 1.5 | 0.13 | 97 | <0.00001 |
| Shenmai | 4 | 282 | 56.57 [19.49, 93.66] | 2.99 | 0.003 | 96 | <0.00001 |
| YXS | 1 | 80 | 27.43 [-5.21, 60.08] | 1.65 | 0.1 | 37 | 0.21 |
| duration |  |  |  |  |  |  |  |
| <=14d | 6 | 501 | 66.57 [25.16, 107.98] | 3.15 | 0.002 | 98 | <0.00001 |
| >14d | 2 | 320 | 27.86 [9.91, 45.82] | 3.04 | 0.002 | 0 | 0.44 |
| LVEF |  |  |  |  |  |  |  |
| EF<=50% | 4 | 339 | 79.79 [36.27, 123.31] | 3.59 | 0.0003 | 96 | <0.00001 |
| EF not limited | 4 | 482 | 31.76 [8.78, 54.73] | 2.71 | 0.007 | 78 | 0.001 |
| 7. MLHFQ | 6 | 436 | -9.68 [-13.67, -5.70] | 4.76 | <0.00001 | 83 | <0.0001 |
| different drug |  |  |  |  |  |  |  |
| SFI | 2 | 124 | -15.65 [-27.58, -3.73] | 2.57 | 0.01 | 90 | <0.0001 |
| Shenmai | 3 | 232 | -5.89 [-8.70, -3.08] | 4.11 | <0.0001 | 63 | 0.07 |
| YXS | 1 | 80 | -9.62 [-15.68, -3.56] | 3.11 | 0.002 | 12 | 0.29 |
| duration |  |  |  |  |  |  |  |
| <=14d | 5 | 356 | -9.99 [-14.64, -5.34] | 4.21 | <0.0001 | 87 | <0.00001 |
| >14d | 1 | 80 | -9.62 [-15.68, -3.56] | 3.11 | 0.002 | 12 | 0.29 |
| LVEF |  |  |  |  |  |  |  |
| EF<=50% | 2 | 120 | -4.71 [-7.22, -2.20] | 3.68 | 0.0002 | 36 | 0.21 |
| EF not limited | 4 | 316 | -11.99 [-17.45, -6.53] | 4.31 | <0.0001 | 80 | <0.00001 |

## Supplementary References

1. Geng, J., Dong, J., Ni, H., Lee, M. S., Wu, T., Jiang, K., et al. (2010). Ginseng for cognition. Cochrane Database Syst. Rev. 12, Cd007769.
2. Lee, H. W., Lee, M. S., Kim, T. H., Alraek, T., Zaslawski, C., Kim, J. W., et al. (2022). Ginseng for erectile dysfunction: a Cochrane systematic review. World J. Mens Health. 40, 264-9.
3. Wang, X., Zhao, Z., Hou, Y., Tang, E., Zhao, G., Wang, S., et al. (2018). Assessment of complementary treatment with Yiqi Fumai lyophilized injection on acute decompensated ischemic heart failure (ACT-ADIHF): rationale and design of a multicenter, randomized, controlled trial. Cardiovasc Drugs Ther. 32, 295-300.
4. Wang, K. (2019). Clinical evaluation study of tonic herbal injections for cardiovascular diseases based on network meta-analysis. [master’s thesis]. (Beijing): Beijing University of Chinese Medicine.
5. Wang, H., and Liang, Y. (2018). China heart failure diagnosis and treatment guidelines 2018. Chin. J. Cardiol. 46, 760-89.
6. Wang, B., Sun, P., Ding, T., and Yin, H. (2016). Statistics on the dosage forms of ginseng in the 2015 edition of the Chinese Pharmacopoeia and analysis of clinical applications. Cardiovasc, Dis. Electron. J. Integr. Trad. Chin. West Med. 4, 171-2.
7. Sun, Y., Liu, Y., and Chen, K. (2016). Cardiovascular pharmacological effects of ginsenosides: progress and reflections. Sci. Sin. (Vitae). 46, 771-8.
